# Dimeric ***Cinchona*** alkaloids

**DOI:** 10.1007/s11030-014-9563-1

**Published:** 2015-01-15

**Authors:** Przemysław J. Boratyński

**Affiliations:** Department of Organic Chemistry, Wrocław University of Technology, Wyspiańskiego 27, 50-370 Wrocław, Poland

**Keywords:** Quinine, Quinidine, Dimer, Trimer, Cinchona, Alkaloid

## Abstract

Nature is full of dimeric alkaloids of various types from many plant families, some of them with interesting biological properties. However, dimeric *Cinchona* alkaloids were not isolated from any species but were products of designed partial chemical synthesis. Although the *Cinchona* bark is amongst the sources of oldest efficient medicines, the synthetic dimers found most use in the field of asymmetric synthesis. Prominent examples include the Sharpless dihydroxylation and aminohydroxylation ligands, and dimeric phase transfer catalysts. In this article the syntheses of *Cinchona *alkaloid dimers and oligomers are reviewed, and their structure and applications are outlined. Various synthetic routes exploit reactivity of the alkaloids at the central 9-hydroxyl group, quinuclidine, and quinoline rings, as well as 3-vinyl group. This availability of reactive sites, in combination with a plethora of linker molecules, contributes to the diversity of the products obtained.

## Introduction

**Fig. 1:**
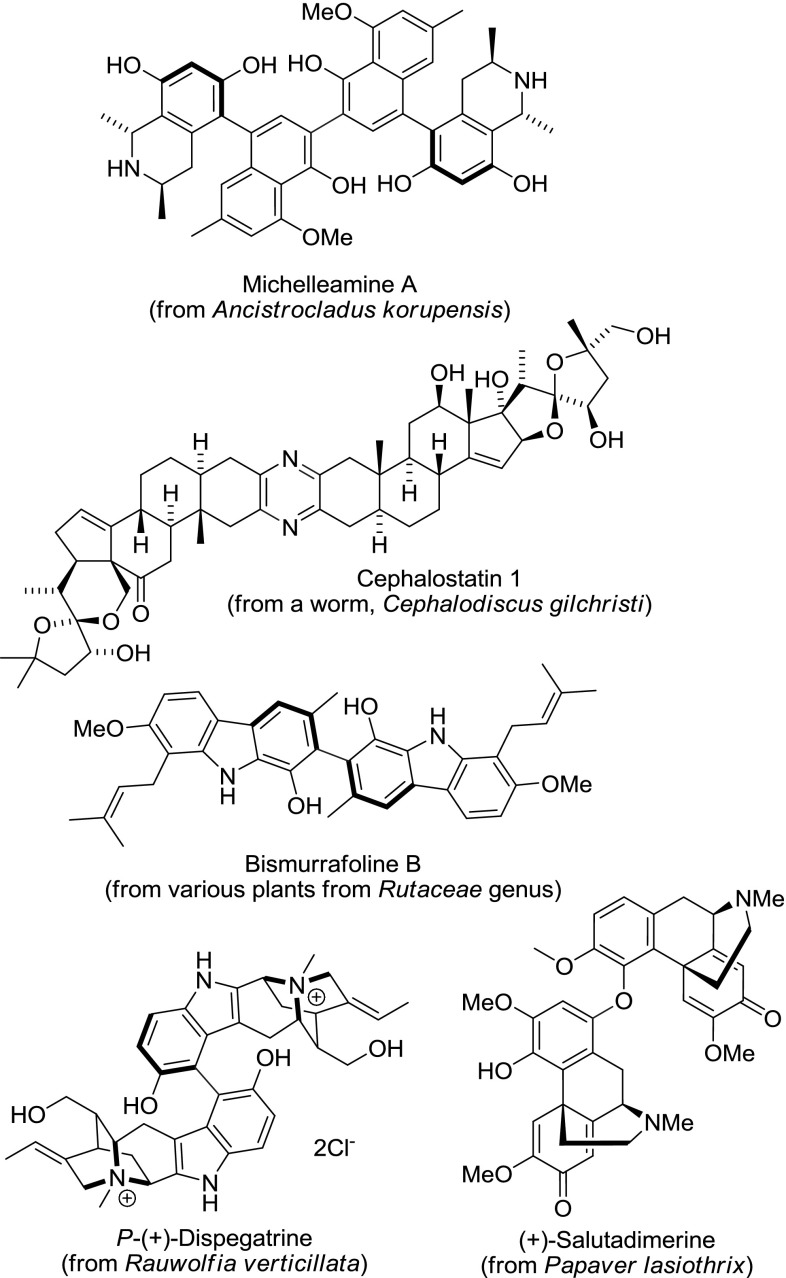
Selected natural dimeric alkaloids

The term *alkaloid* is used for many vastly different nitrogen heterocycles of mostly plant origin. Alkaloids are classified according to the heterocycle and the taxonomy of the species they were isolated from. The natural diversity of the alkaloids is further extended by the presence of numerous dimeric alkaloids (Fig. 1). Some dimers appear as byproducts by coupling of a small portion of the monomers (e.g., salutadimerine), while others are the final products of biosynthesis (e.g., cephalostatin). Alkaloid dimers can exhibit biological activities unrelated to that of the corresponding monomer [1], such as *in vitro* anti-HIV and antimalarial properties of Michelleamine A [2], or fungicidal activity of Bismurrafoline B [3]. Natural alkaloid dimers of different symmetry and heterodimers were isolated (Fig. 1). Additionally, dimers of alkaloids can be synthesized in the laboratory giving rise to a virtually unlimited number of combinations [1].

**Fig. 2:**
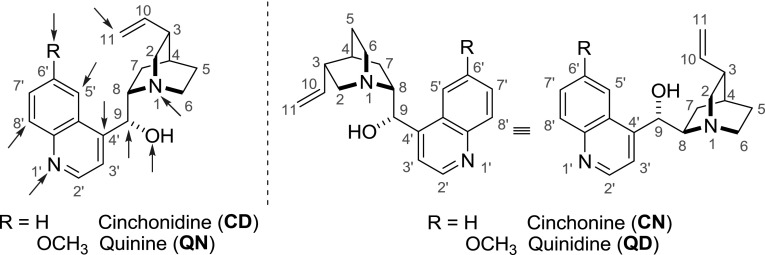
Four major *Cinchona* alkaloids. *Arrows mark* the reactive sites used for dimerization

The bark of various *Cinchona* species contains four major alkaloids, namely quinine (**QN**), quinidine (**QD**), cinchonidine (**CD**), and cinchonine (**CN**) (Fig. 2). These quinoline alkaloids are isolated on an industrial scale in multi-ton amounts. Their structures contain a central hydroxyl group as well as quinoline and quinuclidine rings. The individual alkaloids differ in the configuration at two crucial stereogenic centers (C-8 and C-9, Fig. 2). Quinine has been used for nearly four centuries to treat malaria. Although currently it is largely replaced by newer medicines, such as chloroquine (1947) and artemisinin (1970’s), its therapeutic use is limited to drug-resistant strains. On the other hand, quinidine is often used to treat certain arrhythmias. *Cinchona *alkaloids are also employed in enantioselective synthesis (catalysts, ligands) and separation processes (resolving agents, solid phases, assays) [4]. To date, no dimeric alkaloid in this family has been isolated from a natural source. Nevertheless, many synthetic dimers were made exploiting a few reactive sites in the *Cinchona* alkaloids (Fig. 2).

These synthetic dimers were examined for their biological activities and applicability in asymmetric reactions. For the purposes of medicinal chemistry, the multiplication of the pharmacophore in the dimers could improve binding or cause crosslinking of the biological receptors. The transition from a monomeric to dimeric alkaloid molecule results in accumulation of functional groups confined within a limited space. These features as well as a $C$2-symmetry are often present in effective asymmetric catalysts. Modifications of *Cinchona* alkaloids at the central 9-OH group and at the quinuclidine N-1 atom led to the most effective dimeric catalysts and biologically active compounds.

For the purpose of this review, alkaloid derivatives are labeled with the corresponding alkaloid (**QN**, **QD**... *cf.* Fig. 2), 10,11-dihydroalkaloid (**DHQN**, **DHQD**...), or 9-*epi*-alkaloid descriptor (*e* **QN**, *e* **QD**, *etc.*) followed by a consecutive compound number. It has to be emphasized that some derivatization reactions were reported only for a single alkaloid, while others were exercised on a set of *Cinchona* alkaloids.

## Dimers connected at the central C-9 position

The central 9-OH group offers an attractive site for modification (i.e., etherification and esterification reactions). Alternatively, the hydroxyl group can be replaced with a few other groups (e.g., NH$$_{2})$$ and subsequently used for dimerization.

### 9-Ether-linked dimers

**Fig. 3:**
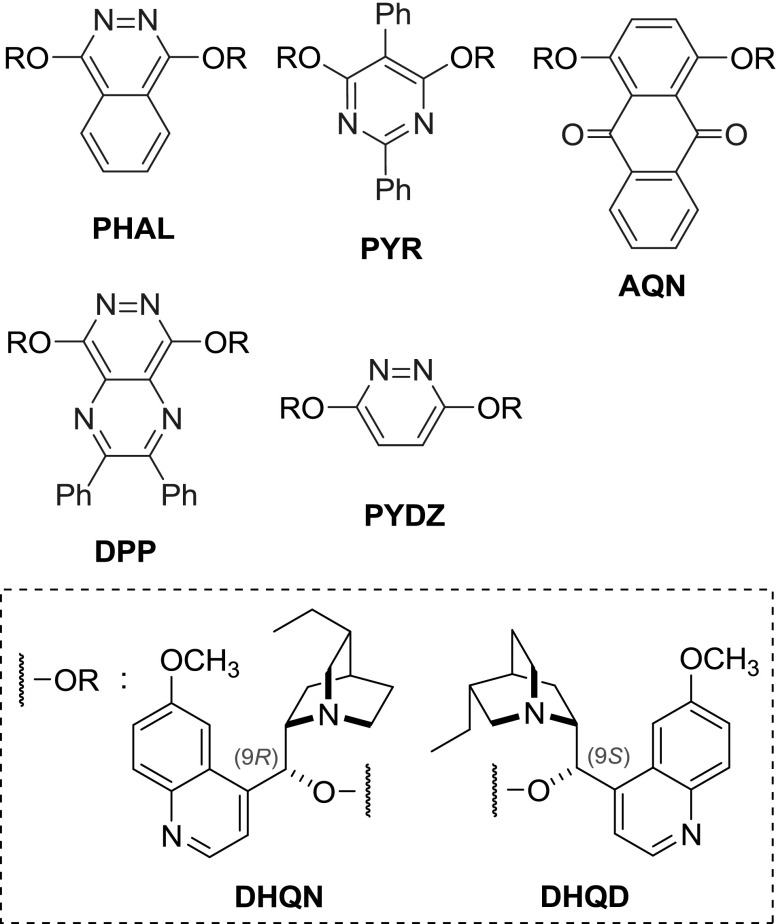
Prominent linkers of dimeric aryl ethers and their common abbreviations

**Fig. 4:**
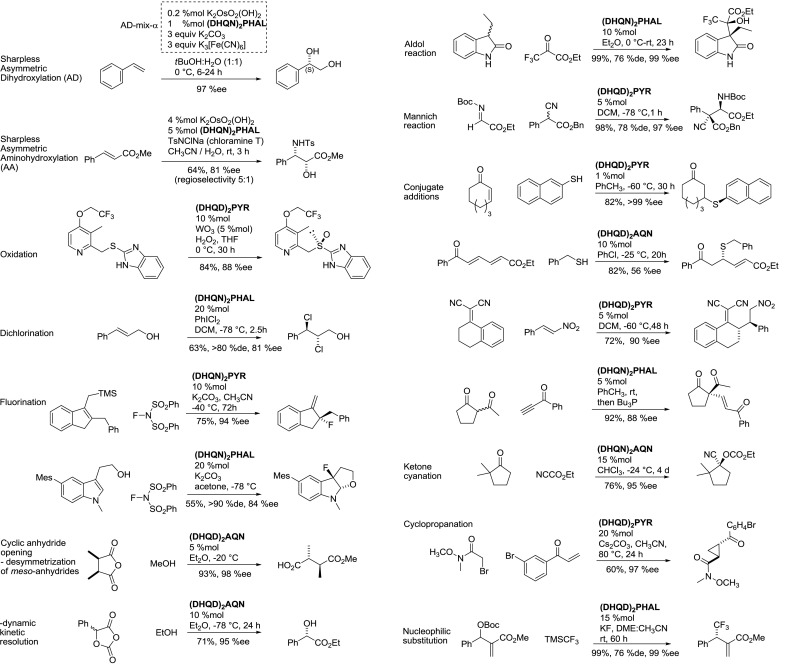
Asymmetric reactions catalyzed by dimeric ethers of *Cinchona* alkaloids

Dimers, in which the *Cinchona* alkaloid units are connected through 9-aryl ethers, represent a class of the most successful ligands for the Sharpless asymmetric dihydroxylation (AD) and related aminohydroxylation reactions (Figs. 3 and 4) [4–8].

The same ligands with tungstate catalyzed enantioselective sulfur oxidation with hydrogen peroxide [9]. Furthermore, numerous applications in metal-free catalysis emerged [4, 10, 11] and made *Cinchona* alkaloid derivatives included to the *privileged* chiral structures [12]. Examples of these asymmetric organocatalytic reactions include (Fig. 4): dichlorination [13], fluorination [14, 15], opening of cyclic anhydrides (including dynamic kinetic resolution) [16, 17], aldol [18] and Mannich-type reactions [19], various types of conjugate addition [20–23], cyanation of ketones [24], cyclopropanation [25], and nucleophilic substitution [26].

In dimeric *Cinchona* aryl ethers (Fig. 3), the linkers are either electron deficient heterocycles or anthraquinones. All of these compounds were obtained through aromatic nucleophilic substitution. Thus, the alkaloid units are mostly in the *para* position with the exception for few *meta* derivatives, but no *ortho*-diethers are known. The lack of such products arises from the reactivity of halo-aryls in the nucleophilic substitution, rather than steric interactions, since heavily substituted pyrimidine derivatives were obtained with relative ease.

#### Phthalazine derivatives

**Fig. 5:**
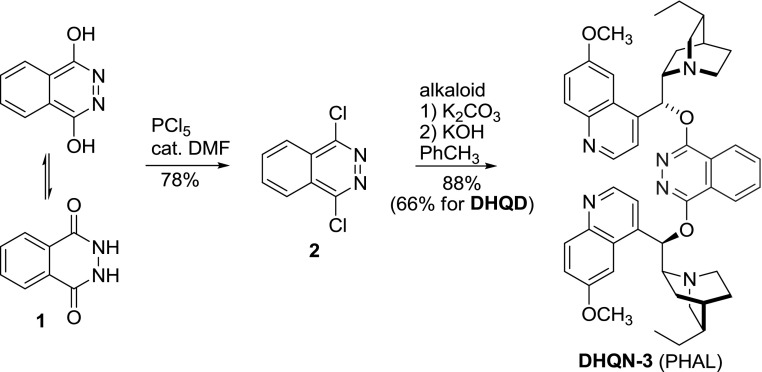
Synthesis of $(\hbox {DHQN})_{2}$PHAL dimer

Up to 1992 there was an incremental progress in osmium-catalyzed asymmetric dihydroxylation reactions, and promising results were obtained with monomeric *Cinchona* alkaloid derivatives in the role of ligands. Then, the discovery of dimeric phthalazine ether ligands (PHAL, **3**) marked an enormous leap for asymmetric synthesis [27, 28]. The respective dimers **3** were obtained from reactions of alkaloids with 1,4-dichlorophthalazine (**2**). The process required basic conditions and azeotropic removal of water with toluene [29]. In an alternative synthesis, the alkaloids were first deprotonated with NaH in DMF and subsequently treated with dichloride **2** [30]. This change in protocol often provided better preparative yields. Although **2** is commercially available, it can be efficiently obtained from phthalhydrazide (**1**), PCl$_{5}$, and a catalytic amount of DMF (Fig. 5) [29].

**Fig. 6:**
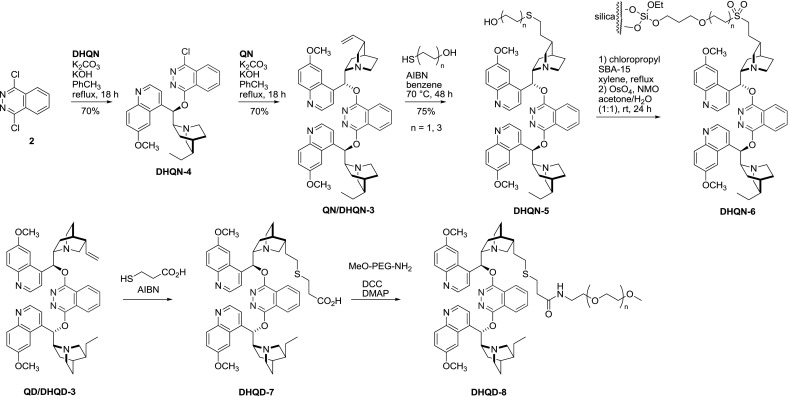
Stepwise synthesis of PHAL-type heterodimers, and their immobilization

Also, a stepwise protocol for the synthesis of unsymmetrical dimers was devised. Equimolar amounts of 1,4-dichloro-phthalazine (**2**) and dihydroalkaloid gave reactive chloroderivative **4** that was used in a subsequent step to 9$O$-arylate another alkaloid. The resulting quinidine-dihydroquinidine and quinine-dihydroquinine heterodimers **3** had a single vinyl group that was used to anchor the molecule to polymer supports using the radical addition of thiols (Fig. 6) [31–33].

**Fig. 7:**
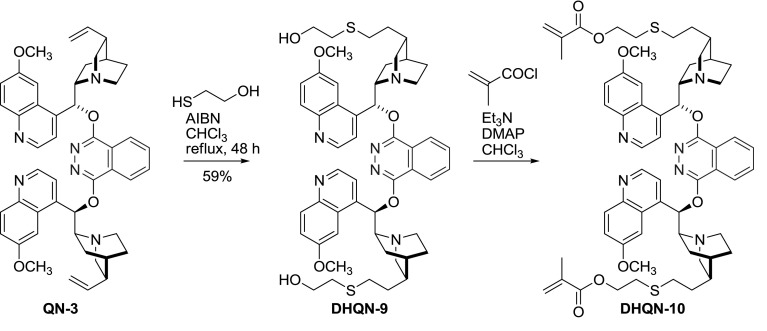
PHAL-dimer derivative for copolymerization

A single reactivity averts crosslinking, and in the cases presented by the authors, also prevents significant distortion of geometry in the parent structure. The same approach was followed to obtain chiral stationary phase additive, by reaction of **QD/DHQD-3** with octadecyl mercaptan [33]. The symmetrical phthalazine dimers **3** were also subject to many subsequent derivatization attempts. These include primarily immobilization, for example, direct copolymerization of quinine-based dimer with methacrylates [34], or copolymerization of more reactive alkaloid-derived acrylate **DHQN-10** (Fig. 7) with styrene/divinylbenzene in suspension [35].

**Fig. 8:**
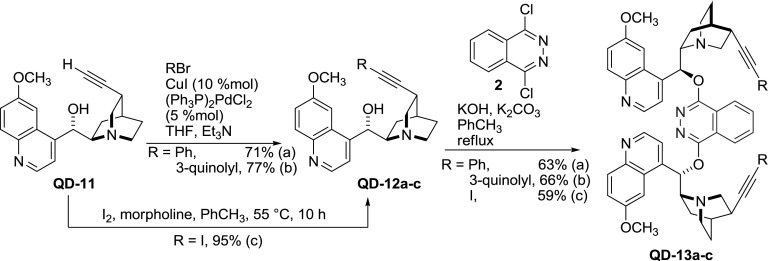
PHAL-type dimers from modified *Cinchona* alkaloids

Apart from simple alkaloids, also their elaborate derivatives were dimerized with phthalazine [36]. Didehydroquinidine (**QD-11**, *vide infra*) was coupled in a Sonogashira reaction with various aryl halides to yield alkaloids with extended carbon scaffold **QD-12a**–**b**. Also, 11-iodinated didehydro-alkaloid **QD-12c** was prepared by addition of iodine to the triple bond of didehydro-alkaloid followed by elimination of HI. These three compounds (**QD-12a**–**c**) were used to obtain respective phthalazine dimers **QD-13a**–**c** (Fig. 8). The yields of the dimerization step were similar to that of unmodified quinidine [37].

**Fig. 9:**
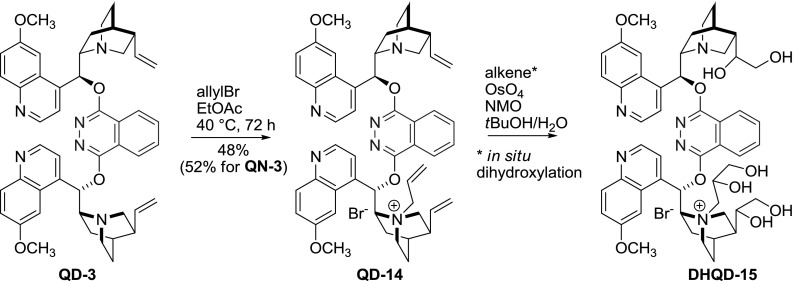
Transformation of PHAL-dimer to hydrophilic species

Under osmium-catalyzed asymmetric dihydroxylation conditions, the two native vinyl groups in *Cinchona* dimers **QN-3**, **QD-3** are transformed to the corresponding tetraols. These products catalyze the AD reaction, although arguably [27] their effectiveness is inferior to **DHQN-3** and **DHQD-3**. Nevertheless, the polar character of these compounds was advantageous for reactions carried in special solvents, including ionic liquids, polyethylene glycol (PEG), and water. Thus, even more polar compounds were obtained by mono $N$1-quaternization with benzyl [30] or allyl bromide. The $N$-allyl ammonium salt **QD-14** was then directly used in the AD reaction. It was transformed *in situ* to water soluble ammonium salt **DHQD-15** having six hydroxyl groups, which facilitated recycling of the ligand through aqueous extraction (Fig. 9) [38]. Also, exhaustive quinuclidine $N$-alkylation was performed on phthalazine dimer **DHQD-3**. The obtained dimeric quaternary salts were not suitable for AD reactions, but were considered for phase transfer catalysis (PTC) [39].

**Fig. 10:**
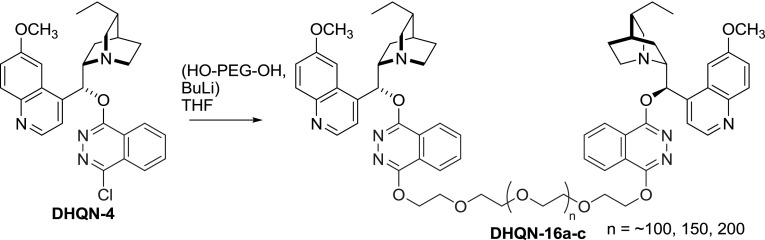
Synthesis of alkaloid phthalazine ether-flanked PEG

Dimers of a different architecture were also obtained in a reaction of polyethylene glycols (PEG) with monomeric dihydroquinine ether **DHQN-4** (Fig. 10). These immobilized soluble ligands **DHQN-16a**–**c** were still successful in aminohydroxylation reactions and could be recycled; however, significant catalyst loading was required [40].

**Fig. 11:**
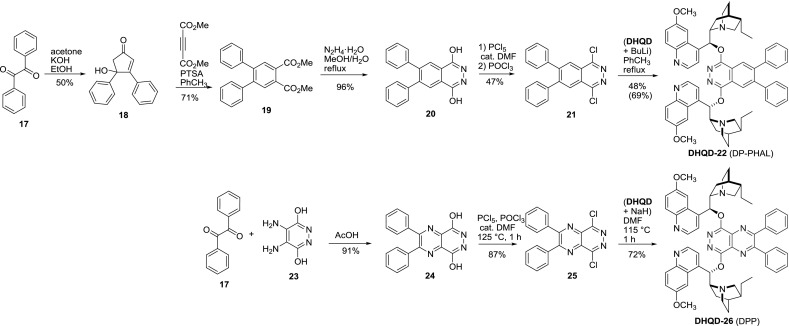
Synthesis of DP-PHAL and DPP-type dimers

Modification at the spacer unit required *de novo* synthesis of the dimers. The analogue of **2** with two pendant phenyl groups, 1,4-dichloro-6,7-diphenylphthalazine (**21**) was obtained in four steps (16 % total yield) from benzil (**17**). Coupling of **21** with both dihydroquinine and dihydroquinidine proceeded to DP-PHAL dimers **DHQN-22** and **DHQD-22** in 69 and 48 % yield, respectively—i.e., by 20 % lower than for the unsubstituted linker **2** [41]. A similar linker with two more nitrogen atoms incorporated into the planar ring system was also applied: 1,4-Dichloro-6,7-diphenyl-pyrazinopyridazine (**25**) [42] was coupled with two dihydroquinidine units providing the respective DPP dimer **DHQD-26** (Fig. 11) [43].

**Fig. 12:**
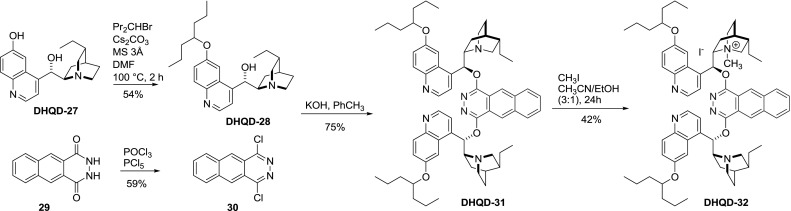
Synthesis of a multiply modified PHAL-type heterodimer

Also, linkers with extended fused ring systems were applied. The synthesis of **DHQD-32** by Corey involves two additional modifications. The alkaloid was first modified at the $6'$-position of the quinoline ring (*vide infra*). Prior to coupling, dihydrocupreidine (**DHQD-27**), a derivative of quinidine with free $6'$-hydroxy group, was $6'O$-alkylated with a series of secondary alkyl bromides [44]. The reactive linker molecule **30** was obtained analogously to phthalazine, starting from naphthalenedicarboxylic acid hydrazide and a mixture of PCl$_{5}$ and POCl$_{3}$ [45]. The coupling of quinidine derivative **DHQD-31** with 1,4-dichlorobenzo[$g$]phthalazine afforded the dimer **DHQD-31** in good yield. Subsequent partial $N$1-quaternization with methyl iodide concluded the synthesis of this highly diversified structure (Fig. 12). **DHQD-32** provided a highly regioselective and enantioselective course of AD reaction of terminal isopropylidene groups in selected terpenoids [44].

#### Pyridazine, pyrazine, and pyrimidine derivatives

**Fig. 13:**
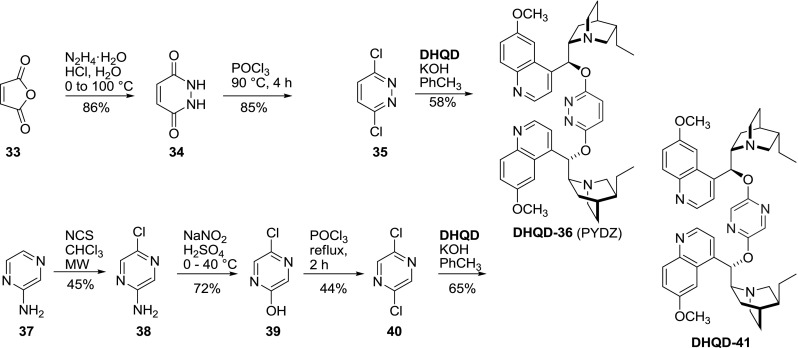
Synthesis of PYDZ and pyrazine-linked dimeric ligands

Shortly after introduction of phthalazine-type ligands by Sharpless, *Cinchona* dimers with two single-ring heteroaromatic linkers were applied by Corey’s group [46]. These included pyridazine **DHQD-36 **(PYDZ) and pyrazine spacers **DHQD-41**. The coupling was accomplished by refluxing the respective 3,6- or 2,5-dichloro heterocycles **35** and **40** with dihydroquinidine in toluene in the presence of a base and the azeotropic removal of water. 3,6-Dichloropyridazine (**35**) not only is commercially available, but can also be obtained in a short and efficient synthesis [47]. On the other hand 2,5-dichloropyrazine (**40**) is more challenging to obtain (Fig. 13) [48, 49].

**Fig. 14:**
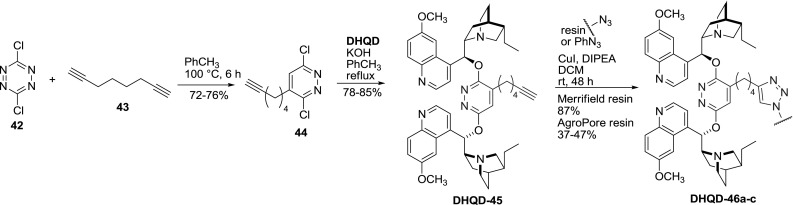
Synthesis of PYDZ derivative reactive in “click” chemistry

Similarly to PHAL-dimers, a few modifications to the original PYDZ structure **36** were made. Double tethered derivatives of **36** are presented in the last section of this article. Dimer **36** was also partially quaternized with 9-anthracenylmethyl group [50]. A pyridazine linker substituted with a short alkyl chain flanked with a terminal alkyne group was also obtained. The functionalized reactive dichloroheterocycle **44 **was obtained via the sequential Diels-Alder and retro-Diels-Alder reactions of dichlorotetrazine (**42**) and 1,7-octadiyne (**43**) in one pot. After the coupling of **44** with dihydroquinidine the terminal alkyne group of the dimer **DHDQ-45** remained reactive in the copper-catalyzed Huisgen 1,3-dipolar “click” cycloaddition (CuAAC) [51]. Thus 1,2,3-triazoles were obtained with various azides including small molecules [52] and polymers (Fig. 14) [51].

**Fig. 15:**
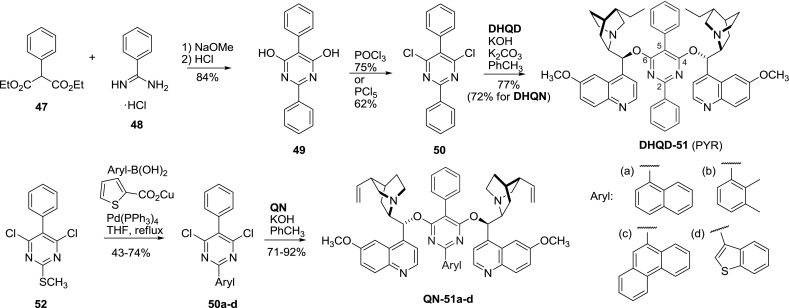
Representative syntheses of PYR-type dimers

Pyrimidine-based dimers **51** (PYR) constitute another important group of ligands, particularly useful in AD of branched olefins. Their major distinction is that the alkaloid units are positioned *meta* instead of *para* to each other. The synthesis again relied on refluxing the dichloroheterocycle **50** with the alkaloid in the presence of a base in toluene and the azeotropic removal of water (Fig. 15). The reactive dichloride **50** was obtained in a two-step procedure starting from the condensation of adequately substituted diethyl malonate **47** and amidine **48**. Subsequent reaction with either POCl$_{3}$ or PCl$_{5}$ gave the required reactive intermediate **50**. An important feature of the pyrimidine scaffold is that 2- and 5- substituted derivatives are often easily accessible. Sharpless obtained dimers with pyrimidine linkers substituted at position 2 and 5 with combinations of phenyl and *tert-*butyl groups [53]. However, groups with greater steric demands at position 2 impede the formation of the dimer, and only monomeric alkaloid derivatives could be obtained from 2-CEt$_{3}$-5-$t$Bu-substituted pyrimidine [54]. In later reports, more differently 2- and 5-substituted and unsubstituted pyrimidine dimers were mentioned [55]. The diversity of the products was further enhanced in a synthesis of several 2-aryl substituted dimers. The commercially available 4,6-dichloro-2-methylthio-5-phenylpyrimidine (**52**) reacted with a series of arylboronic acids in a Suzuki-type reaction. The obtained intermediates with quinine provided the dimers **QN-51a**–**d** in very good yields (Fig. 15) [56]. Also, an analogue of **DHQN-51** substituted at the 2-position of the pyridine with 3,4,5-trimethoxyphenyl group was specifically designed for AD step in a synthesis of a natural product [57]. It was established that for applications in AD the presence of 2-*tert*-butyl is detrimental, while substitutions at 5-position are more tolerated [53]. Nevertheless, such tuning of the catalyst structure with bulky groups improved its performance in an asymmetric Feist–Bénary reaction [55, 56].

**Fig. 16:**
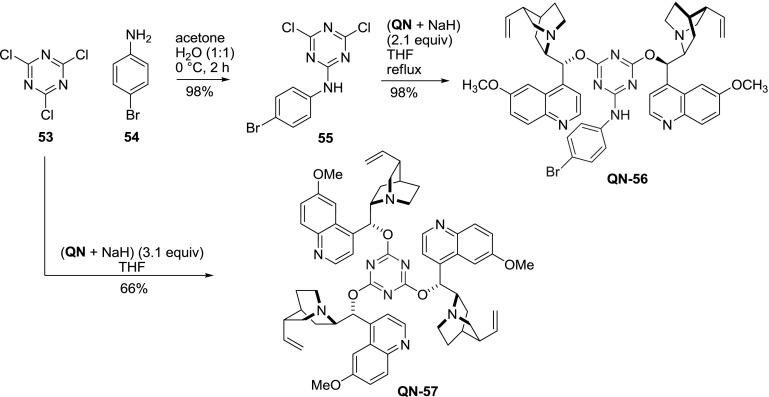
Synthesis of 1,2,3-triazine-linked dimer and trimer

Also, a related spacer with 1,3,4-triazine core was exploited. The synthesis was based on the reaction of inexpensive cyanuric chloride (**53**) with aniline to replace one of the reactive chlorides. Then, dichloride **55** was reacted with the prepared *in situ* quinine sodium salt in THF to provide the respective dimer **QN-56** in nearly quantitative yield. Although the authors used only 4-bromoaniline (**54**), they proposed that a diverse array of products could be obtained using different aniline or amine derivatives. Interestingly, an excess of quinine sodium salt with cyanuric chloride gave $C3$-symmetric trimeric derivative **QN-57** (Fig. 16). However, only dimer **56** showed promise in AD reactions [58].

#### Anthraquinone derivatives

**Fig. 17:**
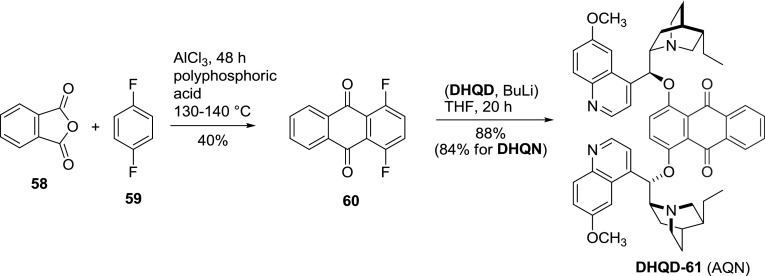
Synthesis of AQN-type dimeric ligands

Apart from the heterocyclic spacers, also the anthraquinone unit was extensively studied. 1,4-Difluroanthraquinone (**60**) was obtained in the Friedel–Crafts reaction of phthalic anhydride with $p$-difluorobenzene. For the coupling, dihydroalkaloid was converted *in situ* into a lithium salt with butyllithium, and then a reaction with difluorocompound **60** yielded the anthraquinone dimers (AQN, **DHQN-61**, **DHQD-61**) in very good yield (Fig. 17) [59]. These ligands are superior in AD of alkenes with aliphatic substituents.

**Fig. 18:**
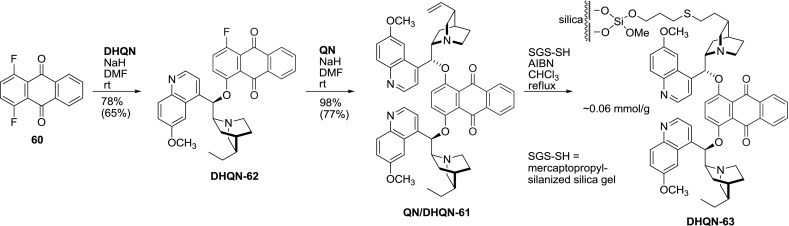
Stepwise synthesis of AQN-type heterodimers, and their immobilization

Similarly to the phthalazine ligands, also a stepwise synthesis was devised. Consecutive reactions of **60** were carried out with alkaloid sodium salts in DMF. This approach, although lower in yield, allowed for the synthesis of heterodimers namely, alkaloid-dihydroalkaloid pair **QN/DHQN-61** suitable for immobilization (Fig. 18) [60].

**Fig. 19:**
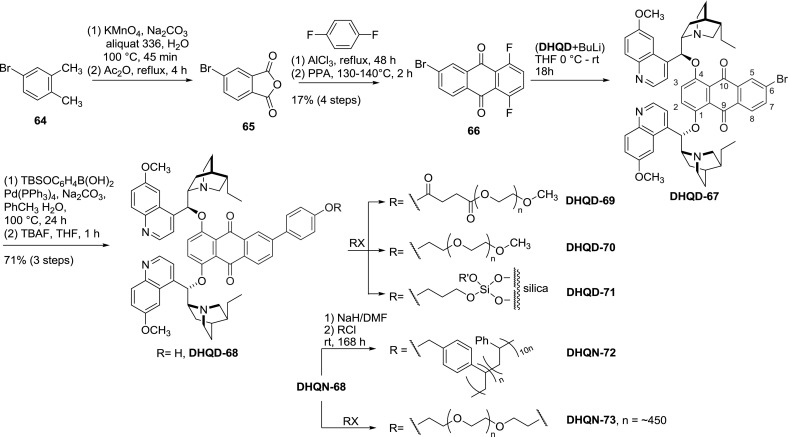
Synthesis of dimers with modified anthraquinone spacer

Other immobilization attempts included addition of thiols to quinine and quinidine homodimers (**QN-61** and **QD-61**) [61]. Also, the spacer was modified to accommodate further transformations. 6-Bromoderivative of anthraquinone **66** was obtained from 4-bromo-$o$-xylene (**64**) in a four-step synthesis. The coupling with the alkaloid afforded 6-bromo derivative of AQN-dimer **DHQD-67**, which was reactive in a Suzuki coupling with TBS-protected 4-hydroxyphenylboronic acid. The silyl ether was cleaved, and the obtained phenol group was exploited to obtain a series of derivatives **69**–**73 **(Fig. 19) [62]. Among these were linear polystyrene [63], silica gel supported material, polyethylene glycol derivatives [62] including a tetramer **DHQN-73** formed from tethered dimeric quinine units [64].

#### 9-Alkyl ethers

**Fig. 20:**
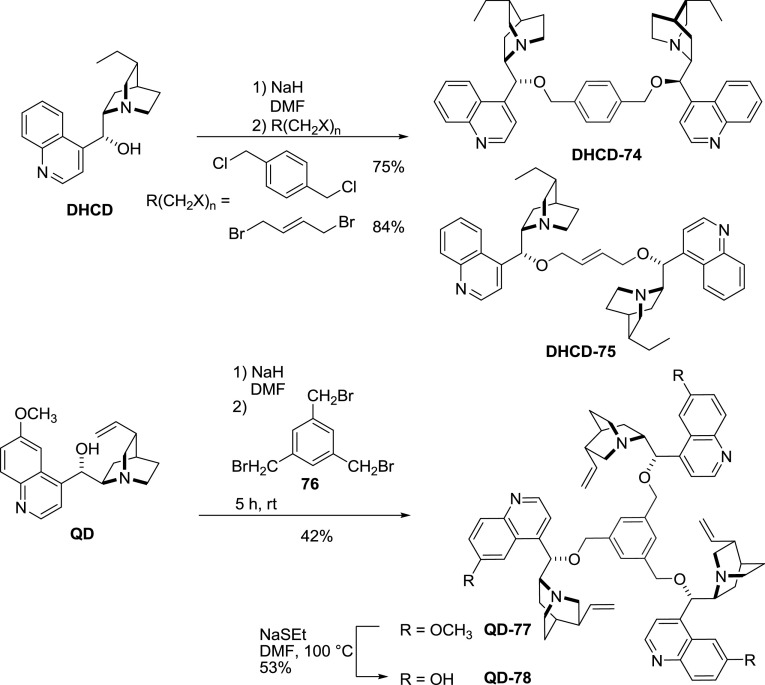
General synthesis of dimeric and trimeric alkaloid alkyl ethers

Dimeric alkaloid alkyl ethers constitute a much less studied group of compounds. Their synthesis is, however, straightforward and involves the Williamson etherification of an alkali metal alkaloid salt and the respective alkyl dihalide. For xylylene-linked dimers, all three isomers: *ortho* [65], *meta* [66], and *para* [67, 68] were reported or mentioned in the literature. The shortest known link was made with $E$-butene, still in very good yield [68]. A trimeric ether **QD-77** was also obtained in an analogous reaction with 1,3,5-tris(bromomethyl)benzene (**76**) (Fig. 20) [69]. Some of the dimeric ethers were converted to polymeric quaternary ammonium salts (*vide infra*) with a series of bis(bromomethyl)arenes and served as effective PTC catalysts [68].

Alkyl ethers are much less suitable ligands for the asymmetric dihydroxylation reaction in comparison to the corresponding aryl ethers. For example, a dimer with $p$-xylylene linker (**DHQD-74**) gave merely 22 %ee in AD reactions where aryl ethers provided excellent enantioselectivity [67]. Nevertheless they showed promise in organocatalytic applications such as asymmetric aminooxygenation of oxindoles (*meta-*xylylene linker) [66].

### 9-Ester-linked dimers

**Fig. 21:**
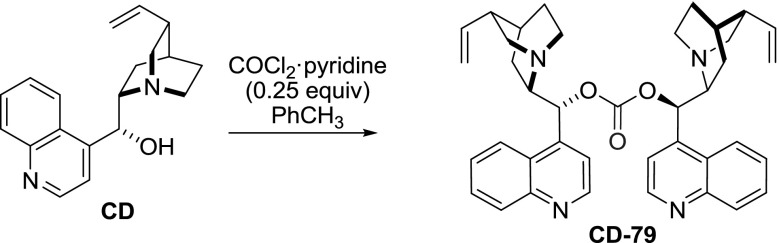
Synthesis of dimeric alkaloid carbonate

The dimeric esters of alkaloids have the longest history of the presented groups of compounds. The carbonic acid diester is known from the patent literature dating to the end of the XIX century (German patent No. DE105666, 1898). In a later published work, the carbonate **CD-79** was obtained in a reaction of excess of cinchonidine with a carefully controlled amount (0.25 equiv) of phosgene (Fig. 21). When the amount of phosgene was increased, an unreactive byproduct, identified then as alkaloid chloroformate, was formed. On the other hand, the carbonate **CD-79** was reported to decompose in water [70].

**Fig. 22:**
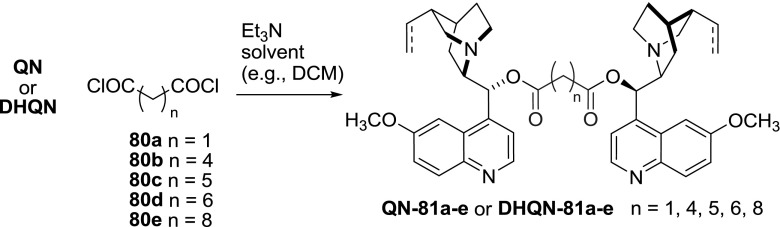
General synthesis of alkaloid dimeric esters

**Fig. 23:**
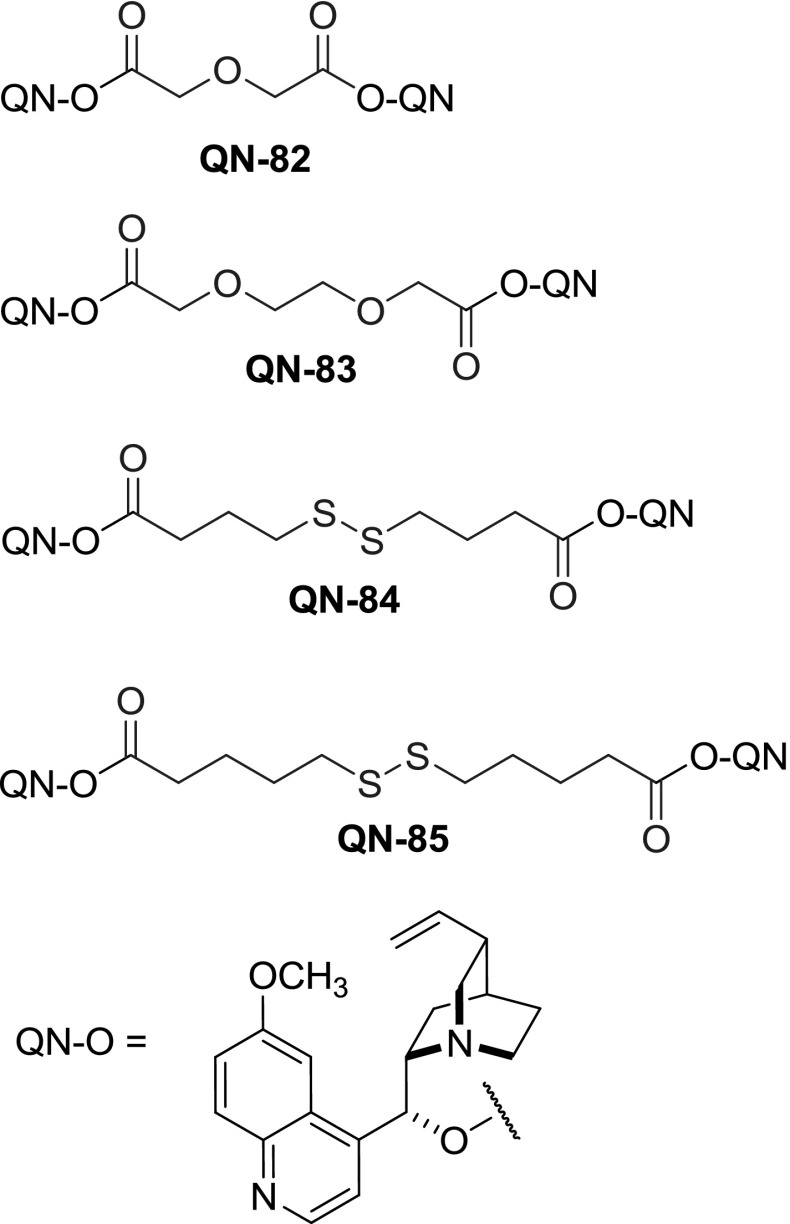
Linear spacers with heteroatoms

Esters of dicarboxylic acids are generally more stable. They were most often obtained in a reaction of dicarboxylic acid chlorides and the alkaloids. Usually the transformation was carried in the presence of a base such as triethylamine [71] and sometimes with catalytic amounts of 4-dimethylaminopyridine (DMAP). The yields, when reported, were above 70 %. The dimers were also obtained in a one-pot procedure, where the starting diacids were first transformed to the corresponding chlorides with thionyl or oxalyl chloride and subsequently coupled with the alkaloids. Alternatively the dicarboxylic acids were activated with a carbodiimide (e.g., EDC); however, this milder method often resulted in poor yields [72]. Following one of these general methods, a relatively large array of dimeric esters was synthesized (Figs. 22, 23). Links were formed from simple aliphatic diacids with 3–10 carbon atoms [71, 72], as well as those with ether (German patent No. DE237450, 1909) and disulfide bonds [72, 73].

**Fig. 24:**
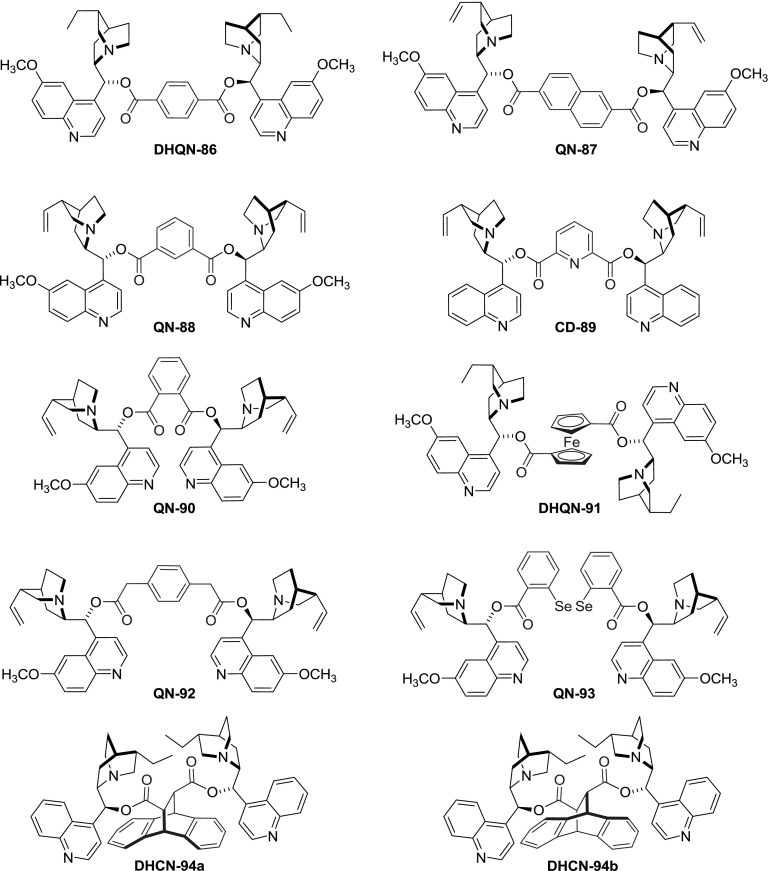
Representative dimer structures with aromatic spacers

**Fig. 25:**
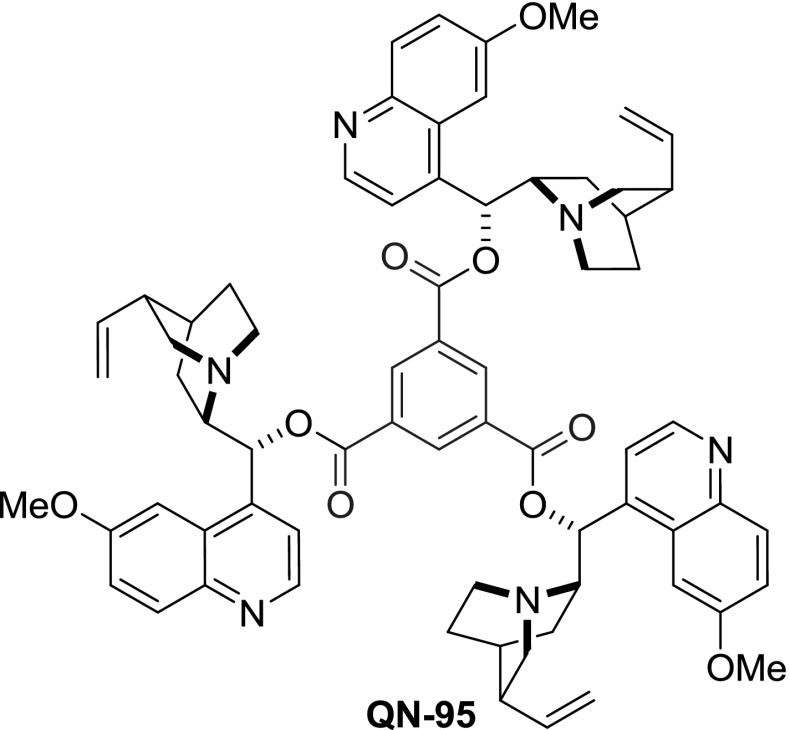
Trimeric *Cinchona* alkaloid ester

Dimers linked with spacers having unsaturated bonds, aromatic rings, and bicyclic scaffolds were also obtained, mostly using corresponding acid chlorides for coupling. *Cinchona* dimers with all of the isomers of benzenedicarboxylic acid were obtained, namely terephthalic [71], isophthalic [74], and $o$-phthalic esters **86**, **88**, and **90**, respectively. Dimeric esters with linkers incorporating heteroatoms were obtained, starting from dichlorides of pyridine 2,6-dicarboxylic acid [75], $2,\!2'$-diselenodibenzoic acid [76], and ferrocene $1,\!1'$-dicarboxylic acid (Fig. 24) [77]. Also, a $C3$-symmetric trimer **QN-95** was formed in a reaction of trimesic acid chloride with quinine (Fig. 25) [71].

A few of the esters were tested in AD reaction. Some, like the ferrocene-linked dimer **DHQN-91** provided only moderate enantioselectivity ($<$61 %ee). In the group of simple diesters, hexadionate **DHQN-81b** turned out an effective catalyst (92 %ee) in contrast to malonate. The most attractive catalytic properties were found for the terephthalate ligand (**DHQN-86**, $>$98 %ee), which in AD of certain substrates outperformed the classic PHAL-type ligand **DHQN-3** [71, 78]. On the other hand, isophthalic ester, while still effective in AD, provided worse enantioselectivity than terephthalates in all the cases studied [74]. In a later study, improved results in AD and AA reactions were achieved for isophthalates and a series of analogous pyridine-linked dimers **89** [79].

**Fig. 26:**
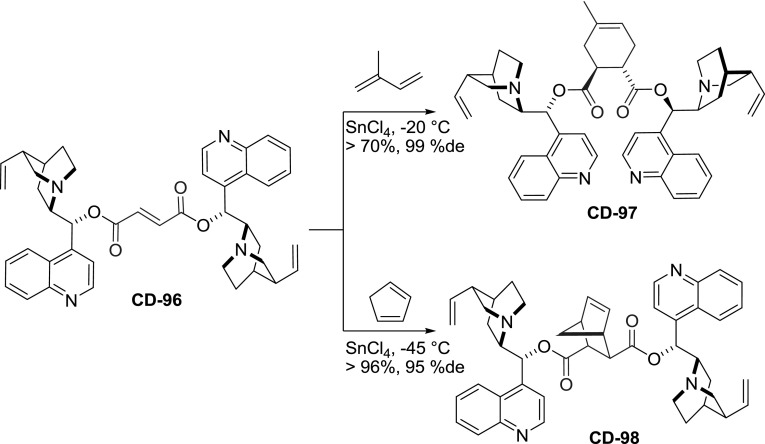
Cyclic linkers obtained in diastereoselective Diels-Alder reaction

Diversity in the linker structure was also introduced by subsequent modifications of an already dimeric molecule. In the reaction of fumaroyl dichloride with cinchonine and cinchonidine, the respective dimeric esters **CN-96** and**CD-96 **were formed in 78–86 % yield [80]. The reactivity of the activated double bond was further exploited in a Diels-Alder reaction with cyclopentadiene and isoprene (Fig. 26). Consequently, a set of cyclohexene- and bicyclic dicarboxylic acid esters **97** and **98** were obtained. These were, however, not viewed as target compounds, instead *Cinchona* alkaloids were used as chiral auxiliaries for the Diels-Alder reaction, and the esters were cleaved afterward. The transformations using dimers provided much improved enantioselectivity compared to that of monomeric esters also used in the study (94–99 % vs. 6–93 %ee) [80]. Interestingly, an inverted sequence of Diels-Alder and acylation reactions was also explored. The enantiomeric diacids, adducts of anthracene and fumaric acid, were converted to acid dichlorides and reacted with dihydrocinchonidine. The products were assayed in asymmetric dihydroxylation of stilbene giving 52–85 %ee. Better results were obtained for ester of 11$R$,12$R$ configuration **DHCN-94b** than the 11$S$,12$S$ diasteromer **DHCN-94a** (Fig. 24) [81].

**Fig. 27:**
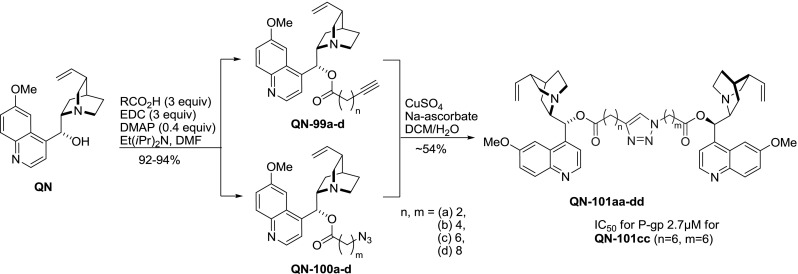
“Click” assembly of quinine esters

Another strategy for the synthesis of dimeric esters of *Cinchona* alkaloids was to first obtain monomeric esters with linker precursors that could be coupled later. In one such approach esters with a terminal alkyne group **99a**–**d** and azido-esters **100a**–**d** of various chain length were formed by carbodiimide coupling. Using these components a combinatorial library of dimers **101aa**–**dd** was obtained in a copper-catalyzed 1,3-dipolar ‘click’ cycloaddition (Fig. 27). In products **101** the linker units are non-symmetric. Since the esterification step is performed separately, well-defined heterodimers could also be obtained. The products were studied for inhibition of P-glycoprotein, and optimum linker length was established at 6 methylene groups at each side of the triazole unit (as in **QN-101cc**) [82].

**Fig. 28:**
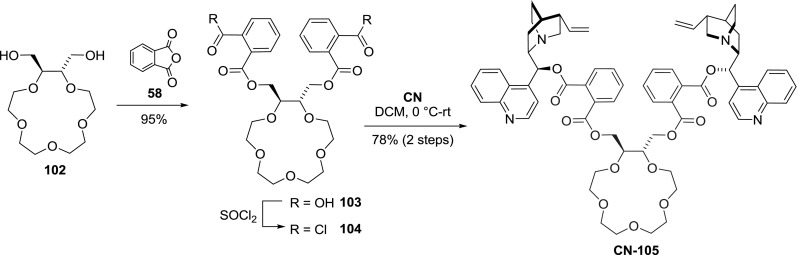
Synthesis of crown ether-linked dimer

Few examples of dimers with more elaborate and functional linkers relevant to supramolecular and coordination chemistry were obtained. One such scaffold incorporated a chiral crown ether. Ether **102 **with two hydroxymethyl groups was modified with phthalic anhydride, and the resulting diacid was converted to acid chloride **104**. Subsequent reaction of **104** with cinchonine gave dimeric ester **CN-105** in 78 % yield (Fig. 28). Although the authors saw potential in the product for phase transfer catalysis, they did not develop the idea further [83].

**Fig. 29:**
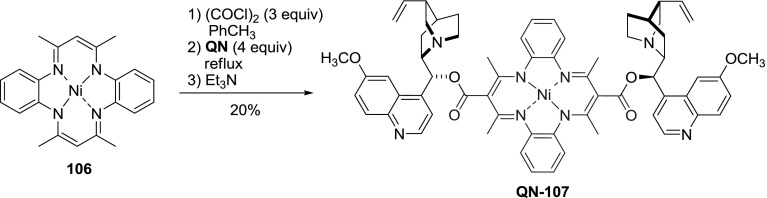
One-pot synthesis of dibenzotetraaza[14]annulene-linked dimer

In a study on the derivatization of annulenes, a one-pot procedure to introduce one or two alkoxycarbonyl groups to nickel dibenzotetraaza[14]annulene complex (**106**) was developed. The sequence of reactions involved Friedel–Crafts acylation with oxalyl chloride, followed by decarbonylation and alkoxydehydrohalogenation [84]. The chirality of the product was assured with nonracemic alcohols, including quinine. The respective dimeric ester **QN-107** was formed in 20 % yield (Fig. 29). This result is similar to that obtained for other explored alcohols [85].

**Fig. 30:**
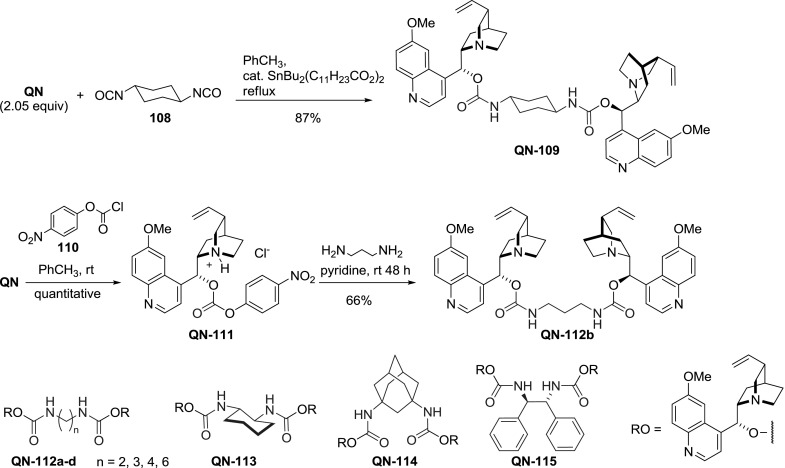
9$O$-Alkaloid carbamate dimers and their synthesis

A series of dimeric carbamates was obtained according to two general protocols [73, 86]. In the first one, the alkaloid was treated with diisocyanate derived from the corresponding diamine. This process was highly efficient furnishing the dimers in 64–87 % yield; however, it was only attempted for the commercially available diisocyanates. Alternatively, a two-step procedure was used: First the alkaloid was converted to an active carbonic ester **QN-111** in a reaction with nitrophenyl chloroformate (**110**). Then, reaction of an excess active ester with diamine gave the corresponding dimeric carbamates in 35–66 % yield (Fig. 30). In both approaches the lowest yields were noted for 1,2-diamine derivatives [86].

Some of the *Cinchona* carbamates were subsequently immobilized on silica (through addition of thiols to the vinyl group) and used as chiral solid phases for anion exchange chromatography of amino acids. The use of dimers, compared to monomeric carbamates, led to longer retention times of the analytes, however, offered no improvement in the enantioselectivity of the separation. Out of the dimeric modifiers of silica gel, the best separation of enantiomers was achieved using the 1,3-adamantyl-linked **QN-114** [86].

The esters and carbamates of *Cinchona* alkaloids were also examined as antimalarial agents. They were tested *in vivo* against drug-resistant *Plasmodium falciparum* and for the inhibition of PfCRT$$^{\mathrm{CQR}}$$—a multidrug resistance transporter protein. Dimers linked with (CH$$_{2})_{8}$$ ester **QN-80e** and (CH$$_{2})_{6}$$ carbamate **QN-112d** (both with the same number of bonds separating two quinine units) turned out to be the most effective [73].

### 9-Nitrogen-linked dimers

**Fig. 31:**
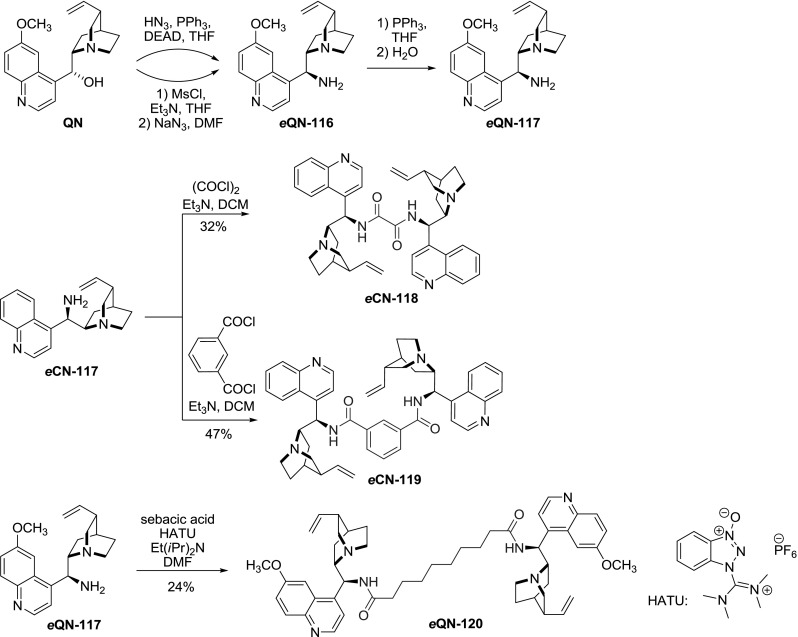
Synthesis of dimeric amides

The 9-hydroxyl group in the *Cinchona* alkaloids can be replaced with an amino group with inversion of configuration at C-9 [87]. The alkaloid undergoes a Mitsunobu reaction with an azide source (e.g., HN3, diphenylphosphoryl azide) to give 9-*epi*-azido-alkaloid **116**. This azide is subsequently reduced with triphenylphosphine to amine **117** (Staudinger reduction). This sequence was often followed as a one-pot procedure. Alternatively, the azido-alkaloid **116 **can be obtained in an S$_{N}2$ reaction from alkaloid methanesulfonate and NaN$_{3}$ in DMF. The primary amino group of the 9-*epi*-9-deoxy-9-aminoalkaloid **117** was then subjected to reactions with various activated linker molecules producing a series of derivatives including amides, ureas, imines, etc. Reaction of oxalyl and isophthalic acid dichlorides with 9-aminocinchonine *e* **CN-117** gave the respective dimers *e* **CN-118** and *e* **CN-119** [88]. Dimeric amide *e* **CN-120** was obtained in a reaction of aminoalkaloid and sebacic acid applying HATU, a standard peptide coupling reagent (Fig. 31) [73]. The oxalyldiamide *e* **CN-118** was tested as catalyst in the diethylzinc addition to benzaldehyde providing only moderate enantioselectivity, though the ee was improved compared to monomeric amide analogs [88].

**Fig. 32:**
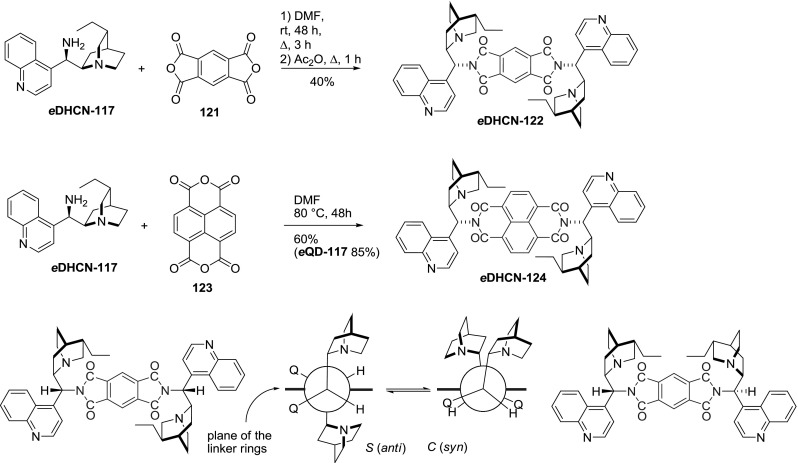
Synthesis of bisimide and a simplified representation of their conformation equilibrium

Gawroński and coworkers obtained dimeric imides *e* **DHCN-122** and **124** in the reaction of *Cinchona *alkaloids with 1,2,4,5-benzenetetracarboxylic anhydride (**121**) and 1,4,5,8-naphthalenetetracarboxylic anhydride (**123**). The initially formed dimeric amide was cyclized to imide *e* **DHCN-122** by heating of the reaction mixture with acetic anhydride. The linear alignment induced by the linker resulted in observable conformer populations, in which the alkaloid units were oriented either *syn* ($$C)$$ or *anti* ($$S)$$ (Fig. 32) [89].

Without an additive, the conformer populations were equal. However, the ratio varied in response to carboxylic and dicarboxylic acids [90]. The prevalent conformation and their equilibria were studied by circular dichroism (CD) [89] and $^{1}\hbox {H}$ NMR spectroscopy. In addition, an analytical system composed of *e* **QD-124 **and bromophenol blue allowed for visual identification of $\alpha$-hydroxycarboxylic acids, as well as their spectrophotometric estimation. This indicator displacement technique allowed for the determination of tartaric acid in wine as well as some differentiation of enantiomers [91].

**Fig. 33:**
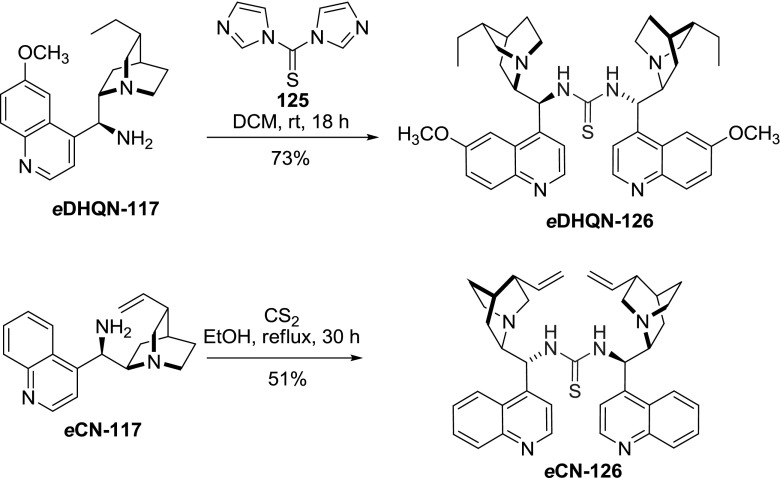
Synthesis of dimeric alkaloid thiourea

A bis-alkaloid-thiourea *e* **CN-126** was obtained in a reaction of CS$_{2}$ with 9-aminocinchonine *e* **CN-117** in 51 % yield [92]. Dimer *e* **DHQN-126 **with the same linker was furnished in a reaction of aminoalkaloid *e* **DHQN-117** with thiocarbonyldiimidazole (TCDI) in DCM in 73 % yield (Fig. 33) [93]. However, other researchers obtained merely 2 % of the identical product *e* **DHQN-126** using THF as a solvent [94]. The monomeric alkaloid thioureas are now well-established bifunctional organocatalysts in asymmetric synthesis [95], while the dimers **126** were found to provide higher level of enantioselectivity in a few cases including dynamic kinetic resolution (DKR) of racemic azalactones (91 %ee with *e* **DHCD-126**) [93] and cooperative sulfonation of enones (76 %ee with *e* **QN-126**) [96]. Also dimer *e* **DHQN-126** had pronounced cytotoxic and cytostatic effects on SH-SY5Y and HL-60 tumor cell lines [94].

A set of ferrocene spacers was applied for the dimerization of *e* **DHQN-117** (Fig. 35). Diamide *e* **DHQN-129** was formed in a reaction between the aminoalkaloid and 1,$1'$-bisfluorocarbonylferrocene, while analoguous urea derivative *e* **DHQN-131** was obtained from 1,$1'$-bisisocyanatoferro-cene. 1,$1'$-Bis(chlorocarbonyl)ferrocene was treated with KSCN in acetone giving a reactive 1,$1'$-bisisothiocyanatocarbonyl intermediate **132**. After solvent exchange, reaction of **132** with aminoalkaloid *e* **DHQN-117** provided dimeric acylthiourea *e* **DHQN-133**. The reported yields for the urea derivatives were very poor (2 %) and were attributed to numerous acylation and polymerization reactions, and tedious purification processes. In the same work, the authors obtained benzenetricarboxylic acid triamide *e* **DHQN-134** in 87 % yield from the respective acid trichloride (Fig. 36). The dimers, in particular *e* **DHQN-129** with the shortest ferrocene link, exhibited pronounced cytotoxic and cytostatic effects on HepG2, SH-SY5Y, HL-60, and MCF-7 human tumor cells [94].

**Fig. 34:**
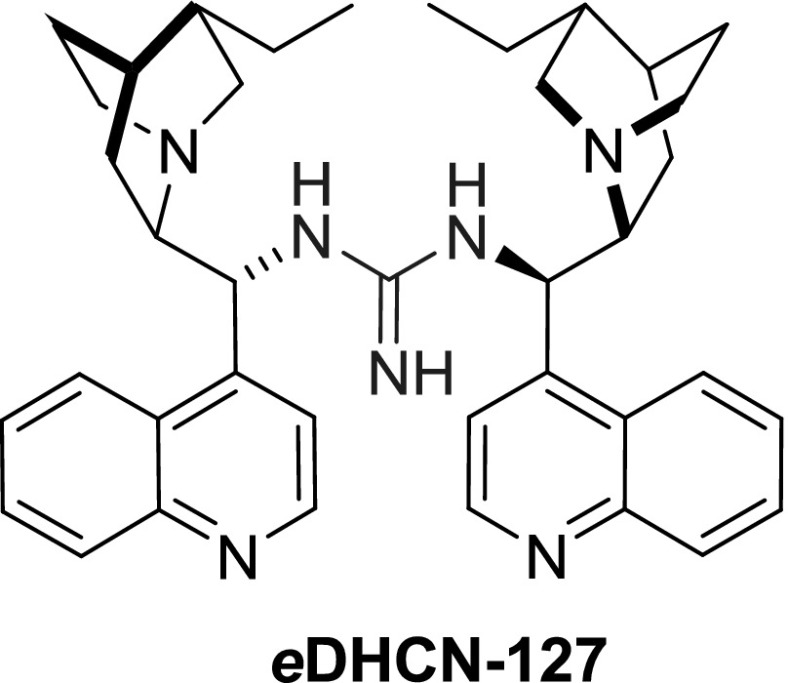
Guanidine-linked dimer

Dimeric guanidine derivative *e* **DHCN-127** was also noted. It was obtained from 9-amino-dihydrocinchonine *e* **DHCN-117** and BrCN (Fig. 34). This dimer was tested in a diastereoselective Henry reaction, but it was not more selective than other catalysts [97].

**Fig. 35:**
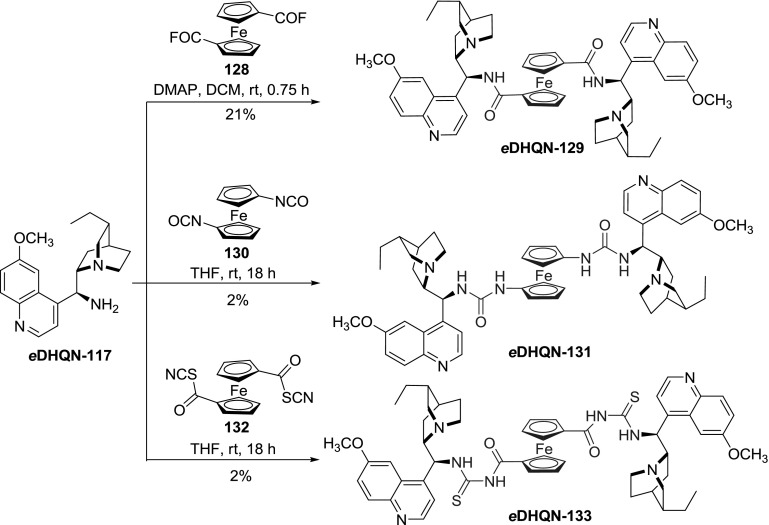
Synthesis of ferrocene-linked dimers: amide, urea, and thiourea

**Fig. 36:**
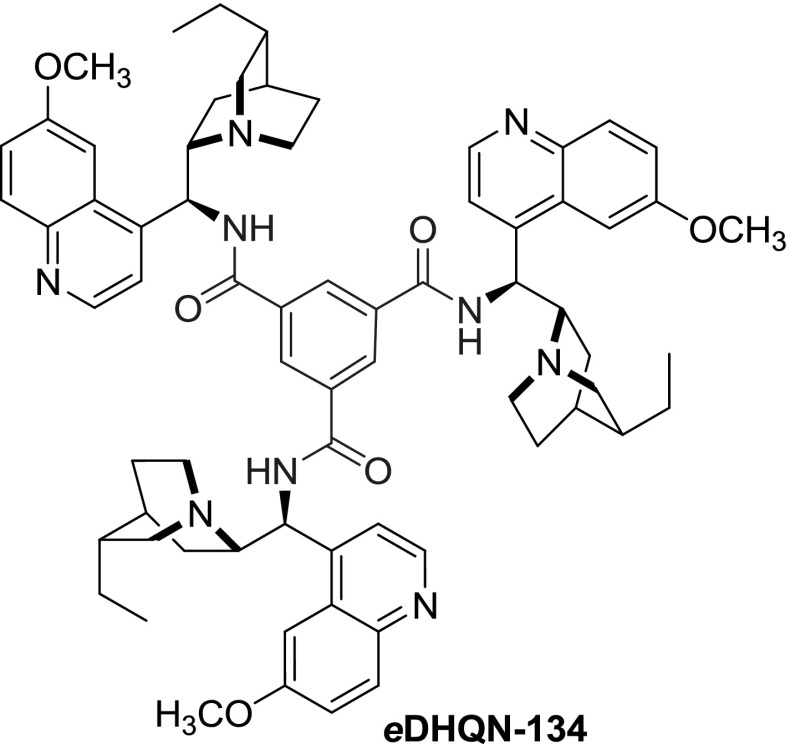
$C3$-symmetric trimeric *Cinchona* alkaloid trimer

**Fig. 37:**
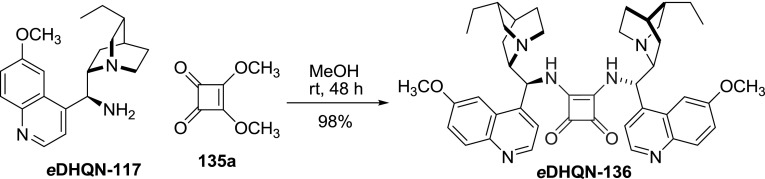
Representative synthesis of dimeric squaramide

Amides of squaric acid (1,2-dihydroxy-cyclobuten-3,4-dione) with *Cinchona* alkaloid units were recently shown to be effective hydrogen bond donors in organocatalysis [98]. The synthesis of dimeric squaramides was straightforward. It entailed mixing 9-aminoalkaloid **117** and squaric acid dimethyl ester (**135a**) in methanol for 24h, while the products **136** precipitated in nearly quantitative yields (Fig. 37) [99]. The dimers were exploited in dynamic kinetic resolution of azalactones. Unlike the monomeric *Cinchona* squaramides, the dimers do not form self-associates [99]. Nevertheless, these two classes of compounds have different application scope, and often unsymmetrically substituted monomeric squaramides were superior catalysts [100].

**Fig. 38:**
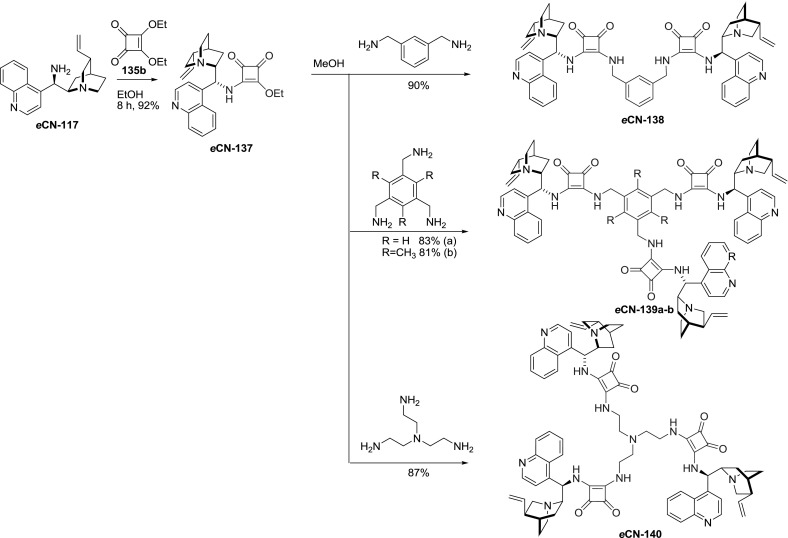
Synthesis of $C2$ and $C3$-symmetric divergently substituted squaramide dimer and trimers

Thus, dimer *e* **CN-138** with two unsymmetrically substituted squaramide units was synthesized, as well as analogous trimers *e* **CN-139** and *e* **CN-140 ** (Fig. 38). First, 9-aminocinchonine *e* **CN-117** was treated with an equivalent amount of diethyl squarate (**135b**), and the intermediate monoester-monoamide *e* **CN-137** was treated with 0.5 equivalents of $m$-xylylenediamine to give a $C2$ symmetric analog of monomeric squaramides. Analogous $C3$-symmetric trimeric compounds were obtained in 81–87 % yield, when instead of diamine, the reactive *e* **CN-137** intermediate was treated with 0.32 equivalents of selected triamines [101]. The trimers *e* **CN-139** and *e* **CN-140 **were effective catalysts in the asymmetric Michael addition of 1,3-dicarbonyl compounds to nitrostyrenes [101] and Friedel–Crafts alkylation reactions [102].

Although a few monomeric alkaloid sulfonamides are known [103], the only example of the corresponding dimers (with $m$-benzenedisulfonamide linker) appears in the patent literature (Chinese patent No. CN103570708, 2013).

**Fig. 39:**
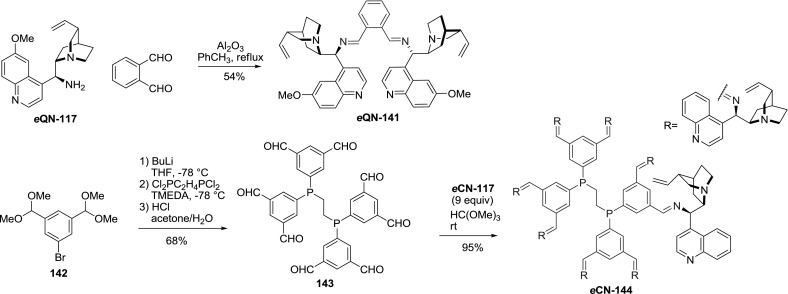
Synthesis of dimeric and dendrimeric imines

Few Schiff bases obtained from the aminoalkaloids **117** and aromatic aldehydes were described. The reaction of aminoquinine *e* **QN-117** with phthalic aldehyde gave rise to dimeric imine *e* **QN-141** in 54 % yield. The efficiency of a single step corresponds to the yields achieved with monoaldehydes under similar conditions (75–81 %) [104]. Also, a dendrimeric Schiff base *e* **CN-144** incorporating eight alkaloid units was obtained. The first generation dendrimeric aldehyde **143** was obtained from 5-bromo-1,3-bis(dimethoxymethyl)benzene (**142**) through halogen-lithium exchange, reaction with bis(dichlorophosphino)ethane (75 %), and subsequent hydrolysis of dimethyl acetal (95 %). The condensation of aldehyde **143** with 9-aminocinchonine *e* **CN-117** using trimethyl orthoformate proceeded in nearly quantitative yield (Fig. 39) [105].

### 9-Carbon- and 9-sulfur-linked dimers

**Fig. 40:**
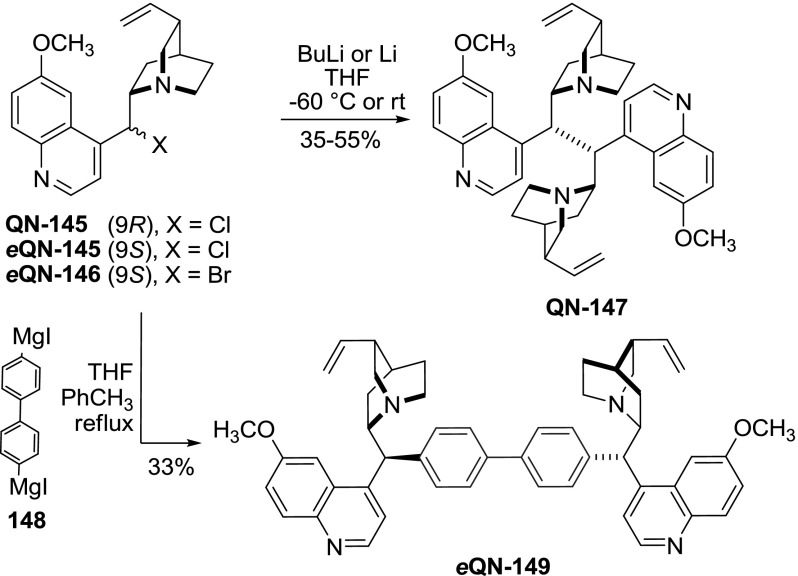
Dimers bound by C-9 carbon–carbon bonds

**Fig. 41:**
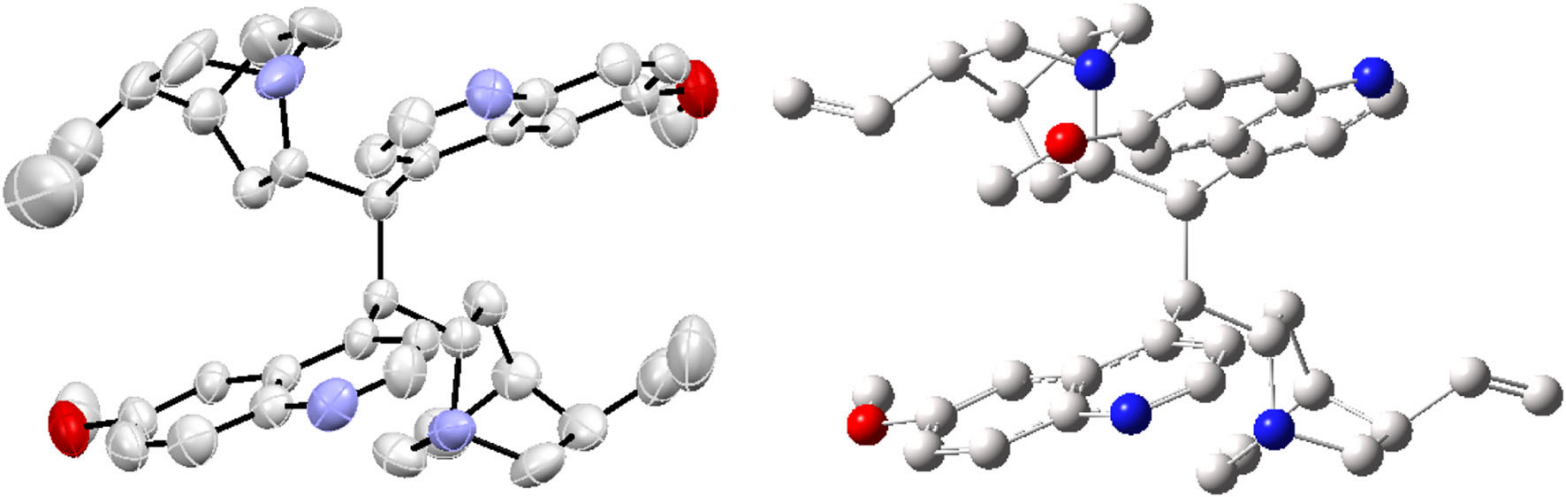
Atropisomers of the dimer **QN-147**, X-ray structure (*left*) and DFT calculated structure for $C1$ rotamer (*right*)

In our research we demonstrated the formation of dimer **QN-147** in which the alkaloid units are connected with a direct chemical bond between two C-9 carbon atoms. This product was obtained by treating quinine-derived 9-halides **145**–**146 **with lithium or butyllithium in THF (Fig. 40). The same isomer of the product (9$$R)$$ was obtained from both 9$$R$$- and 9$$S$$- halides: **QN-145** and *e* **QN-145**, respectively. The most likely explanation for this process was the transient formation of a 9-radical (either direct, or through oxidation of carbanion), followed by radical recombination. The dimers **QN-147 **and **DHQN-147 **are sterically hindered, thus *anti* and *syn* atropisomers (arising from rotation of the quinoline ring, Fig. 41) were separated. The rotational barrier was estimated at ca. 23 kcal/mol in solution [106]. The reaction of organomagnesium reagents with 9-haloquinine [107] was shown to result in a stereoconvergent Würtz-type coupling (the similarity of this process with the direct dimerization to **QN-147** is only superficial, since the products had different configuration at C-9) [108]. The extension of this approach with divalent Grignard reagent **148** gave the respective dimer *e* **QN-149** in moderate yield (Fig. 40) [106].

**Fig. 42:**
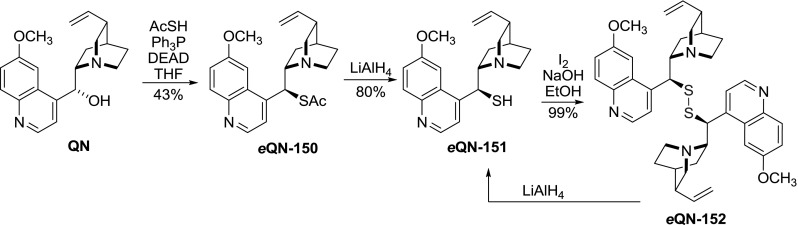
Representative synthesis of 9-disulfide dimer

Also, a set of dimeric 9-disulfides was obtained in a sequence of Mitsunobu, reduction, and oxidation reactions. Seven different alkaloids including **CD**, **QN**, **QD**, **DHQN**, **DHQD**, as well as 9-*epi*-quinine (*e* **QN**), and 9-*epi*-quinidine (*e* **QD**) reacted giving the corresponding dimers **152** in 24–45 % yield with inversion of configuration. It is noteworthy that the oxidative dimerization step was the least demanding. The authors also regarded dimerization as a means for transient protection of the thiol group in **151**, which could be cleanly regenerated with LiAlH$_{4}$ (Fig. 42) [109].

## N1-Qarternary ammonium salts

**Fig. 43:**
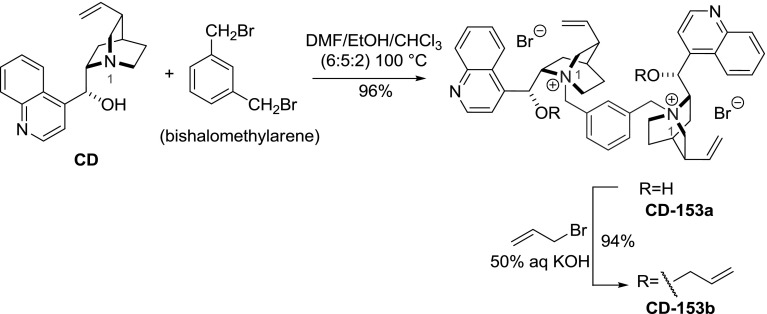
General synthesis of dimeric ammonium salts

Quaternary ammonium salts of *Cinchona* alkaloids were applied in the 1980’s in the asymmetric synthesis under PTC conditions providing decent level of enantioselection. $C_{2}$-symmetric quaternary ammonium salts derived from binaphthalene emerged as even more effective catalysts in 1999 [110]. Shortly thereafter, highly efficient $C2$-symmetric dimeric *Cinchona* alkaloid quaternary ammonium salts were developed [111]. Park and Jew obtained dimers **CD-168a**, **CD-153a**, **CD-169a** in a direct reaction of excess cinchonidine with *ortho*, *meta*, and *para*-xylylene dibromides, respectively, in a mixture of solvents (DMF/EtOH/CHCl$_{3}$, 6:5:2 v/v). The obtained dimers were then alkylated at the 9-hydroxyl group with allyl bromide. The preparative yields were in the range of 90–94 % over two steps (Fig. 43) [111].

**Fig. 44:**
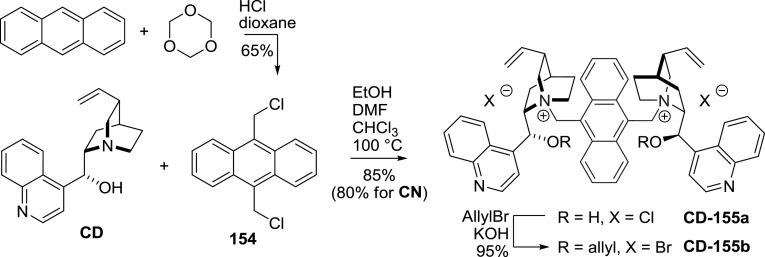
Synthesis of dimethylanthracene-linked dimer

**Fig. 45:**
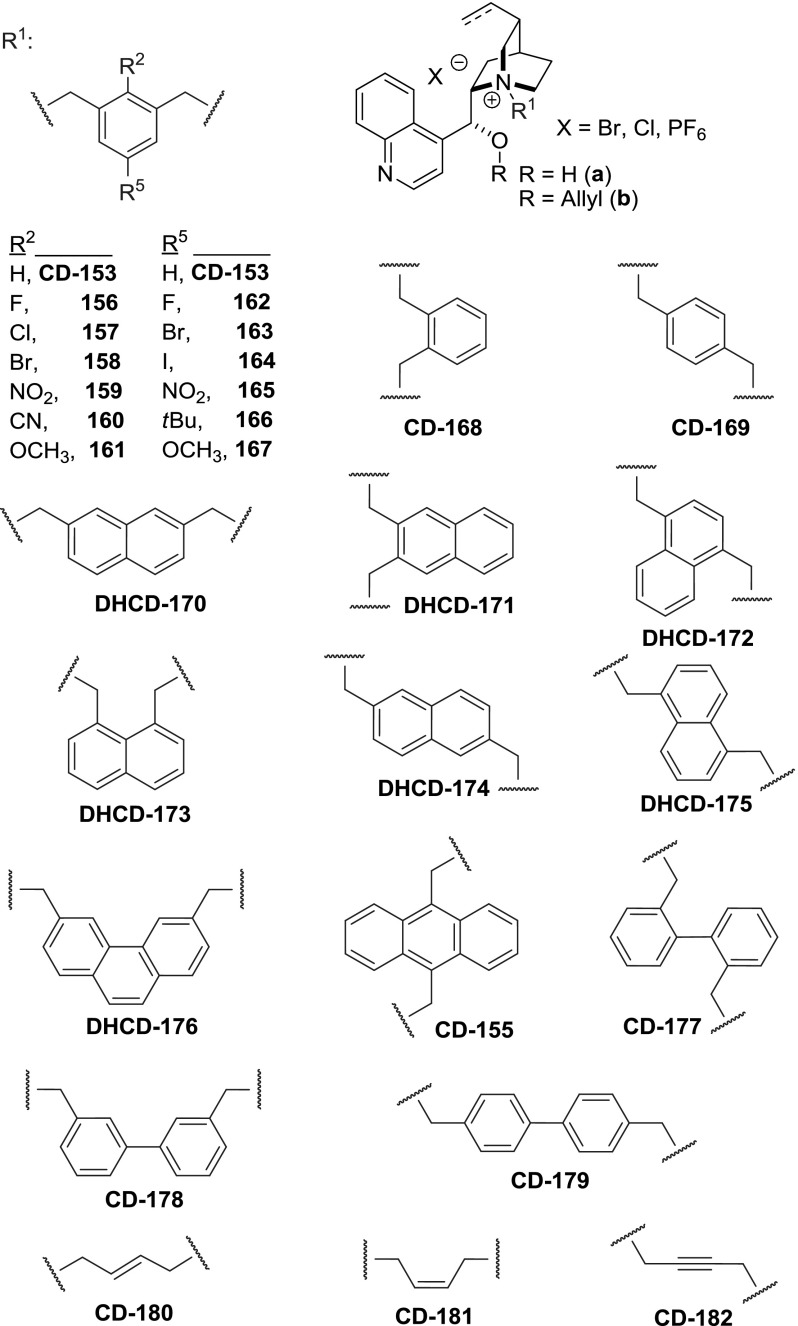
Linkers in dimeric *Cinchona* quaternary ammonium salts

In Park’s subsequent development of catalyst **CD-153b**, a dozen of analogs (**CD-156b**–**167b**) were obtained from differently 2- or 5- substituted $m$-xylylene dibromides in 85–95 % yield [112]. In later works, a quinine analogue was also obtained [113]. The same group synthesized dimers with an extended ring system of the linker, as in naphthalene derivatives **DHCD-170**–**175**. The appropriate reactive halides were obtained from dimethylnaphthalenes through radical bromination with NBS (88 % yield for 2,7-derivative). Reaction of the dibromides with 2.03 equiv of dihydrocinchonidine and dihydrocinchonine and subsequent 9$O$-allylation gave the respective dimers **170b**–**175b** in 90–95 % yield [114]. Further expansion of the ring system was done by the Najera group, who introduced a 9,10-dimethylanthracenyl linker [115]. The required bis(chloromethyl)anthracene (**154**) was obtained from anthracene, paraformaldehyde, and HCl [116]. The reaction of **154** with cinchonidine proceeded with slightly better yield than with cinchonine. One of the obtained dimers was subsequently allylated (Fig. 44) [115]. Also dimers **CD-177**–**179** incorporating a 4,$4'$- [117], 3,$3'$-, and 2,$2'$-dimethylbiphenyl linkers as well as **DHCD-176** with 3,6-dimethylphenanthrene unit were obtained [39]. Apart from the benzyl-type linkers, also chains of $E$- and $Z$-butene as well as butyne were applied in the dimers **CD-180**–**182** (Fig. 45) [39].

**Fig. 46:**
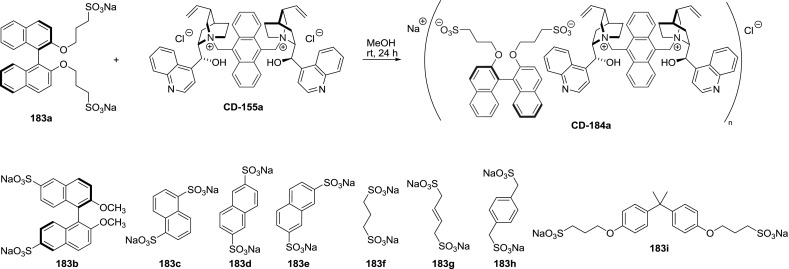
Ionic polymer of dimeric *Cinchona* quaternary ammonium salt and structures of disulfonates

The dimeric quaternary ammonium salts **153**, **155**, and **169** were also converted to ionic polymers (e.g., **CD-184a**) with adequate disulfonates **183a**–**i**. The obtained materials were insoluble in water and most organic solvents (Fig. 46). Nonetheless, these polymers were effective in asymmetric transformations and were easily recovered from the reaction mixture [118, 119].

**Fig. 47:**
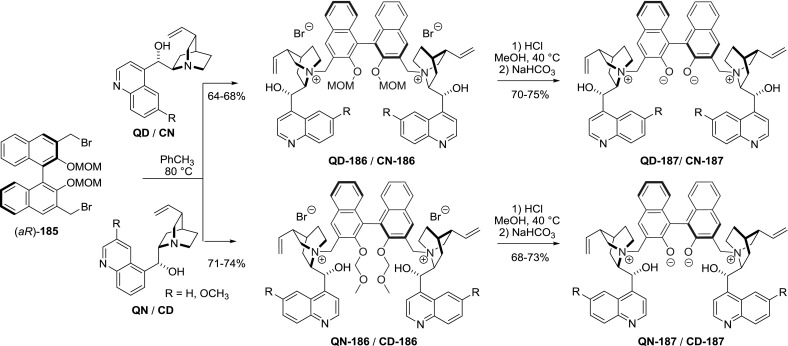
Synthesis of dimeric betaines

**Fig. 48:**
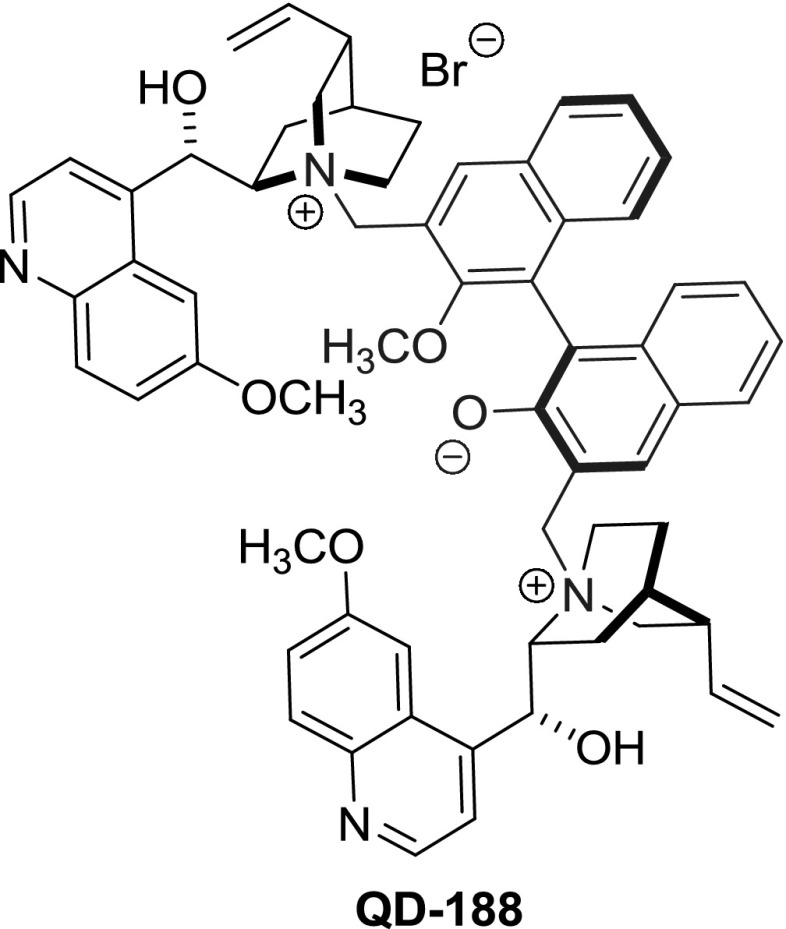
Example of unsymmetrical dimer

Internal quaternary ammonium salts (i.e., betaines) where both cationic and anionic centers are present within a single molecule are also known. They differ in acid–base properties with the compounds described previously, in which there is no covalent bond between the oppositely charged species. The Gong group obtained a set of zwitterionic dimers **187** with a binaphthophenolate linker starting from all major *Cinchona *alkaloids and two axial enantiomers of BINOL [120]. The reactive MOM-protected 3,$$3'$$-bis(bromomethyl)-BINOL derivative **185** was obtained in 5 steps from commercially available BINOL in 68 % yield [121]. The reaction of enantiomeric (*aR*)-dibromide **185** with the alkaloids afforded the respective dimeric ammonium salts **186** in 64–74 % yields. The coupling was slightly more efficient for the alkaloids of quinine configuration (i.e., 8$$S$$,9$$R)$$. Then, the protecting MOM groups were removed producing betaines **187** in 68–75 % yield (Fig. 47). Diastereomeric dimer (*aS*)-**QD-187** was obtained from quinidine and (*aS*)-BINOL derivative (*aS*)-**185** in overall 70 % yield. The authors also obtained species **QD-188** with a net-positive charge, using monomethyl BINOL ether (Fig. 48). The obtained dimeric betaines **187**–**188** were tested as organocatalysts in Mannich reaction of azalactones and aliphatic imines. Dimeric betaine (*aR*)-**QD-187 **was particularly successful providing 96–98 %ee [120].

**Fig. 49:**
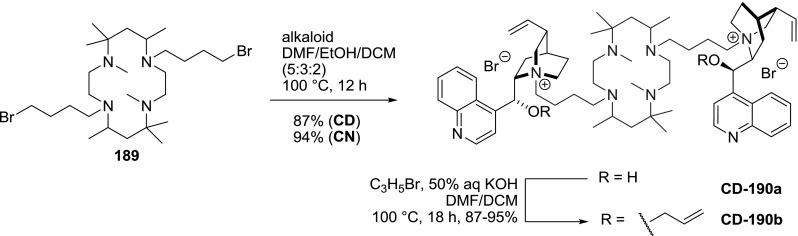
Synthesis of macrocycle-based dimers

Few dimeric *Cinchona *alkaloid quaternary ammonium salts incorporate functional linkers relevant to supramolecular chemistry, such as macrocyclic amine **190**, calixarene **193**, and polyethylene glycol **195**. Siva and Murugan obtained di(bromobutyl)tetraazacyclotetradecane derivative, which was subsequently used for double $N$1-quaternization of both cinchonine (94 %) and cinchonidine (87 %). The obtained dimeric products **190a** were subsequently 9$O$-allylated to give **190b** in high yield (Fig. 49). In the original paper there are, however, some discrepancies between the reported spectral data and structures of **189**–**190** [122].

**Fig. 50:**
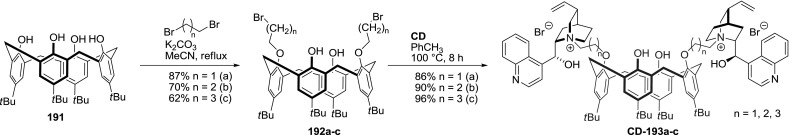
Synthesis of calix[4]arene-linked dimers

Similarly, linkers of various lengths incorporating the calix[4]arene scaffold were synthesized. The reactive dihalides **192a**–**c **were obtained from 4-*tert*-butylcalix[4]arene (**191**) and $\upalpha$,$\upomega$-dibromoalkanes [123]. The dihalides **192** were then used to $N$1-alkylate cinchonidine furnishing dimers **CD-193a**–**c**. The yield of the coupling increased with the separation between the alkaloid units in 86–96 % range (Fig. 50) [124].

**Fig. 51:**
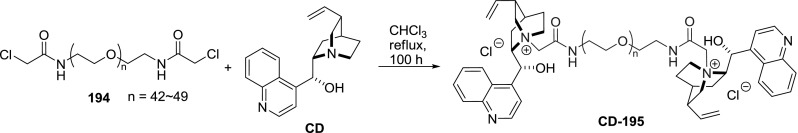
Synthesis of PEG-linked dimers

Polyethylene glycol (PEG2000) was also used in the role of a linker [125]. For this purpose, PEG was converted to a reactive intermediate **194** by introduction of terminal chloroacetamide groups [126, 127]. A subsequent reaction with the alkaloids (**CD**, **QN**, and **CN**) in refluxing chloroform for 4 days afforded the respective dimers **195 **(Fig. 51) [125].

**Fig. 52:**
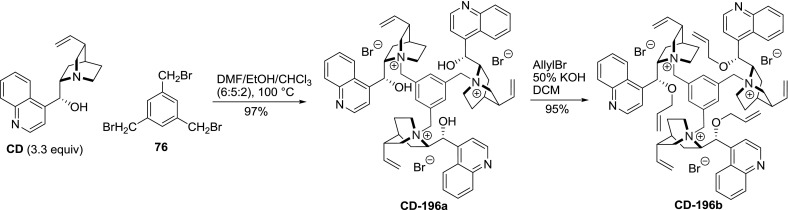
Synthesis of $C3$-trimer

**Fig. 53:**
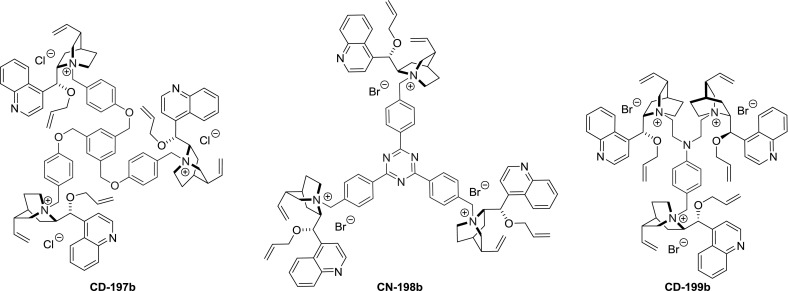
$C3$ and $C1$-symmetric trimeric *Cinchona* alkaloid ammonium salts

It has to be noted that in addition to the dimers, the $C1$ and $C3$-symmetric trimers were obtained. The reaction of $\upalpha$,$\upalpha$’,$\upalpha$”-tribromomesitilene (**76**) and cinchonidine proceeded in nearly quantitative yield and was not impeded by steric interactions. Subsequent 9$O$-allylation gave **CD-196b** in high yield (Fig. 52) [128]. Also, symmetric and non-symmetric trimers **197**–**199** with farther separated alkaloid units were obtained in 79–88 % yield (Fig. 53) [129–131].

Quaternary ammonium salts of *Cinchona* alkaloids were most often employed in asymmetric phase transfer catalysis (PTC), which is useful, for example, for the synthesis of nonracemic amino acids [110, 132]. Phenylalanine derivatives can be obtained through enantioselective benzylation of glycine benzophenone imine esters under PTC conditions. This reaction serves as a benchmark for various catalysts (Table 1) [133].

**Table 1:** Benzylation of $N$-diphenylmethylene glycine *tert*-butyl ester catalyzed by quaternary ammonium salts

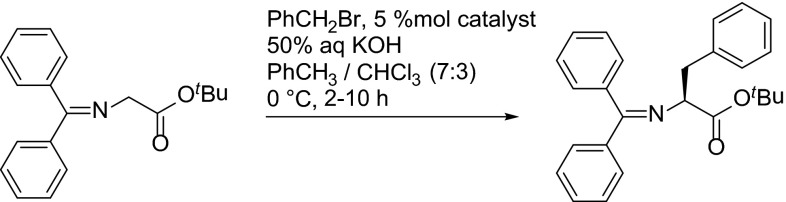			
Catalyst	Time, h	Yield, %	Ee, % (S)
**DHCD-170b**	0.5	95	97
**DHCD-156b**	6	94	96
**CD-156b**	6	93	94
**DHCD-172b**	2	92	91
**CD-153b**	2	91	90
**CD-155a**	6	88	86
**DHCD-175b**	3	90	86
**CD-179b**		93	84
**CD-169b**	4	92	80
**CD-175b**	3	92	80
**DHCD-174b**	3	90	79
$O$-allyl-$N$1-benzyl-cinchonidinium bromide	2	92	75
**CD-155b**	1	84	70
**DHCD-173b**	10	82	44
**DHCD-171b**	10	88	36
**CD-168b**	3	90	31

**Fig. 54:**
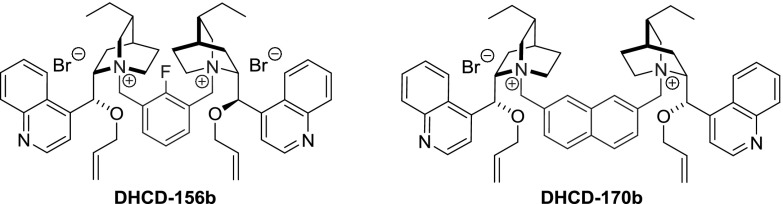
The most effective 1st and 2nd generation Park’s and Jew’s catalysts for the alkylation of Schiff bases under PTC conditions

The first generation of Park’s catalyst **CD-153b**, with *meta*-xylylene linker applied in the PTC benzylation of glycine imine outperformed the monomeric *Cinchona* catalysts both in terms of enantioselectivity and reactivity. On the other hand, application of the isomeric dimer with *ortho*-xylylene linker gave poor ees. The allylation of the 9-hydroxyl in the catalysts often significantly improved the enantioselectivity in the PTC transformation [111]. Study of analogues of **CD-153 **revealed that 2-fluorine atom in the xylylene linker as well as 10,11-hydrogenated alkaloid unit further improved the enantioselectivity [112]. A highly hindered trimer **CD-196b** provided high enantioselectivity $(94\,\%\hbox { ee at } -20^{\circ }\hbox {C})$ at a cost of reactivity [128] The second generation Park’s catalyst **DHCD-170b** with 2,7-naphthyl link was one of the most efficient and enantioselective catalysts for the benzylation of glycine imine under PTC conditions providing 97 %ee at $0^{\circ }\,\hbox {C}$ and 1 %mol loading [114]. This catalyst is commercially available and can be acquired from major reagent suppliers (Fig. 54).

Functional linkers in the dimeric quaternary ammonium salts, in some cases improved their application scope or facilitated the recycling of the catalyst. The macrocyclic dimer **CD-190b** was claimed to be more suitable for PTC reactions carried in low base concentration [122]. Incorporation of PEG within the linker facilitated recovery of the PTC epoxidation catalyst **CD-195** [125].

**Fig. 55:**
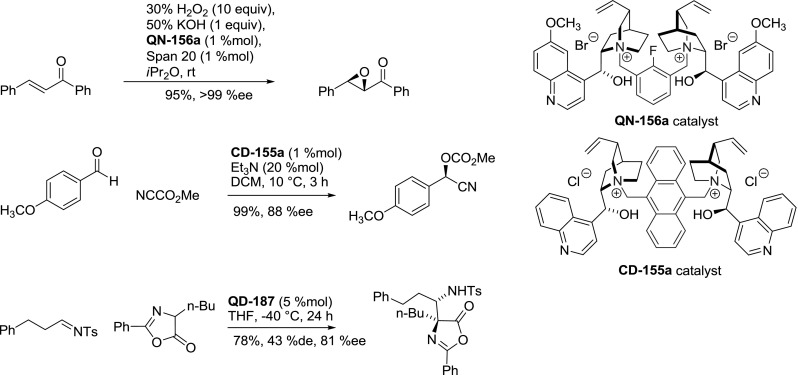
Asymmetric reactions catalyzed by dimeric quaternary ammonium salts of *Cinchona* alkaloids: epoxidation of enones, cyanoformylation of aldehydes, and Mannich reaction

Unfortunately, in many of the studied transformations the replacement of cinchonidine with pseudoenantiomeric cinchonine units resulted in more or less noticeably lower enantioselectivity and conversion. Another major concern in asymmetric catalysis is that the success of a particular catalyst structure in one reaction (e.g., PTC alkylation of glycine imine) does not necessarily translate to other asymmetric processes. For example, epoxidation of enones required a catalyst with free 9-hydroxyl group in the alkaloid unit, and the best results were obtained for quinine analogue **QN-156a**. Moreover, this epoxidation failed to proceed enantioselectively when using analogous monomeric catalysts [113]. Cyanation of aldehydes gave good enantioselectivities with **CD-155a** [134] and so did the Mannich reaction of azalactones catalyzed by **QD-187** (up to 99 %ee for adequately modified reactants) (Fig. 55) [187].

## 3-Vinyl group

**Fig. 56:**
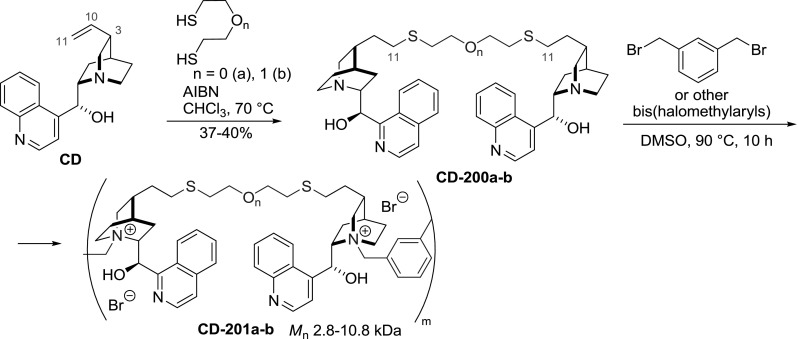
Synthesis of dimer and polymer with dithiol linker

The vinyl group of *Cinchona* alkaloids is an attractive site of derivatization and was often used to couple the alkaloid (and even their dimers) to solid support. This was often achieved by a ‘click’ thiol-ene radical addition. The corresponding reaction of dithiols (butane-1,4-dithiol, and 2-mercaptoethyl ether) and cinchonidine led to dimeric products **CD-200a**–**b** in fair yields [135]. The dimers were subsequently polymerized by tethering at the quinuclidine nitrogen atom (*vide supra*) producing an array of polymers **CD-201** (Fig. 56). These were assayed in the asymmetric benzylation of glycine imine under PTC conditions providing 71–88 %ee.

**Fig. 57:**
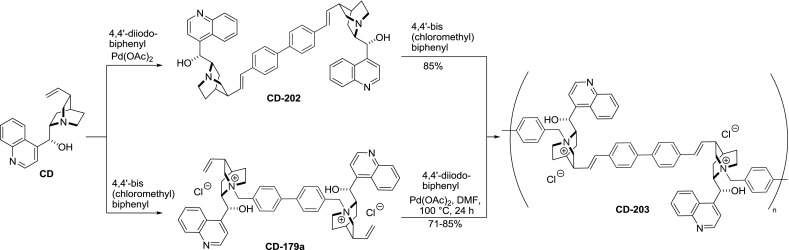
Synthesis of dimers and polymeric materials using the Mizoroki-Heck reaction

Similar polymers, tethered alternatively by quinuclidine nitrogen and vinyl groups, were developed using the Mizoroki-Heck reaction at the alkaloid vinyl groups. First, the dimer **CD-202** was obtained in a palladium-catalyzed reaction of cinchonidine and 4,$4'$-diiodobiphenyl and subsequently was polymerized by quaternization with 4,$4'$-bis(chloromethyl)biphenyl. Polymers **CD-203** were effective in the benzylation of $N$-diphenylmethylene glycine *tert*-butyl ester. Surprisingly, when using an inverted reaction sequence, i.e., polymerization of the dimeric quaternary ammonium salt **CD-179a** in a Heck reaction, the obtained polymeric material **CD-203** had superior catalytic qualities (Fig. 57) [136].

**Fig. 58:**
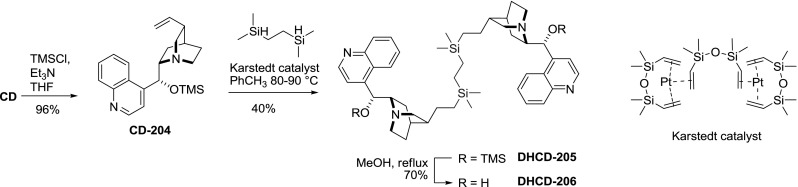
Synthesis of silicon-linked dimers

Cinchonidine dimer **CD-205 **tethered at the vinyl groups with silicon linker was also synthesized. First the 9-hydroxyl group of cinchonidine was transiently protected with TMS ether. Then, hydrosilylation of the ether **CD-204** with bivalent silane (1,2-bis(dimethylsilyl)ethane) was performed applying Karstedt’s catalyst. The coupling gave dimer **DHCD-205** in moderate yield, similarly to reactions of **CD-204** with other bulky mono-silanes. Finally, the TMS group was removed giving **DHCD-206** (Fig. 58). The dimer **CD-206** was applied for modification of Pt/Al$_{2}$O$_{3}$ catalyst surface for asymmetric hydrogenation of ethyl pyruvate and phenylpropanedione. Unfortunately the enantioselectivity was lower than for unmodified cinchonidine (62 vs. 84 %ee) [137].

**Fig. 59:**
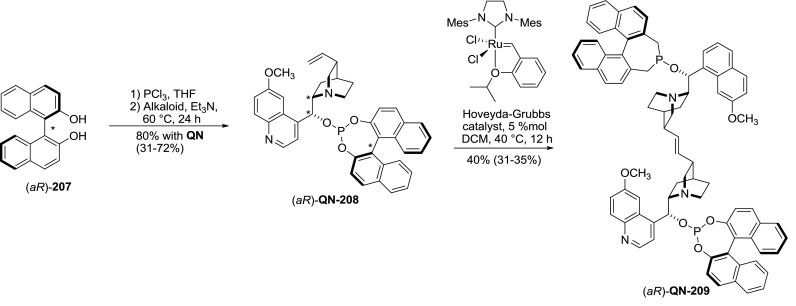
Metathesis dimerization of *Cinchona* alkaloid derivatives (for starting materials of different configuration yields are given in parentheses)

The terminal vinyl group is also reactive in alkene metathesis reactions. This approach was used to obtain a series of dimeric phosphite ligands derived from BINOL and *Cinchona* alkaloids. The phosphite esters **QN-208**, **QD-208**, and **CN-208** were obtained in a one-pot reaction from BINOL (of both *aR* and *aS* configurations) and the corresponding alkaloids. The metathesis reaction of **208** with Hoveyda-Grubbs catalyst produced dimers **209** which were isolated in fair yields (Fig. 59). The authors also obtained libraries containing heterodimers by combining different stereoisomers of the alkaloid phosphites **208** in the metathesis reaction. Dimers **209** along with crude metathesis mixtures were subsequently used in asymmetric iridium-catalyzed asymmetric hydrogenation of $\upalpha$,$\upbeta$-unsaturated carboxylic acids. Out of the studied compounds, the dimer (*aR*)-**QN-209 **provided up to 92 %ee and outperformed the initial monomer ($\Delta$ee 8–32 %) [138].

**Fig. 60:**
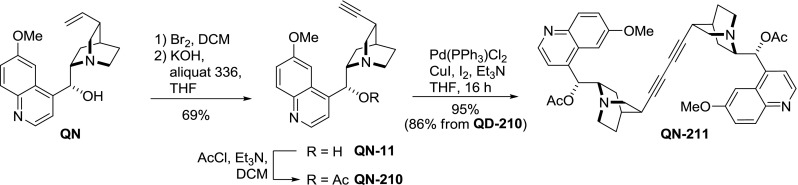
Palladium-copper-mediated dimerization of didehydroalkaloids

The vinyl group in the alkaloids can also be converted to a terminal alkyne by bromine addition followed by two HBr elimination reactions [139, 140]. The alkynes **QN-11** and **QD-11** (didehydroalkaloids) were then directly coupled in a Sonogashira-type oxidation reaction. With iodine as an oxidant, the yields were good (71–72 %) and further improved when 9$O$-acetylated didehydroalkaloids **210** were used (86–95 %; Fig. 60) [36].

**Fig. 61:**
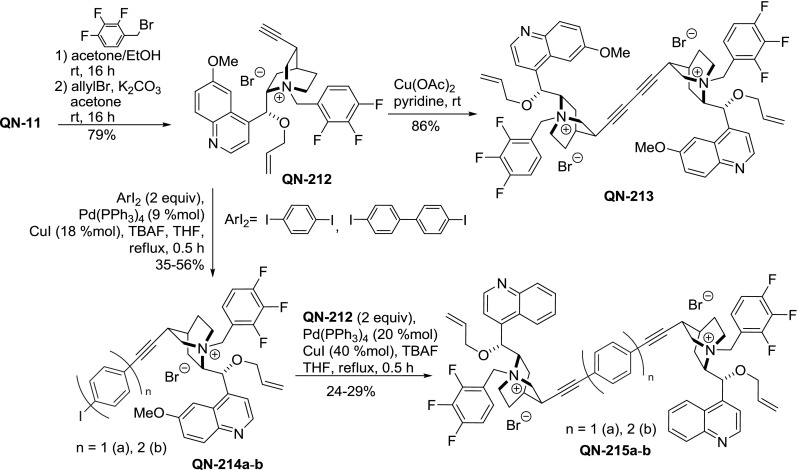
Dimerization of quaternary ammonium salt

Direct dimerization of **210** using typical Glaser coupling (copper(I) salt/O$$_{2})$$ gave merely 15 % yield [36]. In contrast, a similar homocoupling of didehydroalkaloid quaternary ammonium salt **QN-212** with copper(II) salt (Elington reaction) proceeded smoothly giving dimer **QN-213** in 89 % yield. Also, Sonogashira coupling of the alkaloid alkyne **QN-212** with aryl diiodides gave dimers **215a**–**b** with phenylene and biphenylene linkers, respectively. Unfortunately, the authors found that the dimerization cannot be performed as a one-pot reaction, instead a stepwise protocol had to be followed using excess of reagents at each coupling step: First **QN-212** was coupled with 1,4-diiodobenzene or 4,$$4'$$-diiodobiphenyl giving iodoaryl derivatives **QN-214a **and **QN-214b**, respectively. The subsequent coupling with **QN-212** required significant catalyst loading only to proceed in low yields (Fig. 61). The products **QN-213** and **QN-215a**–**b** were tested in the asymmetric aldol reaction under PTC conditions. Optimum performance was reported for the phenylene-linked dimer **QN-215a**, although only moderate enantioselectivity was achieved [141].

## Quinoline ring

**Fig. 62:**
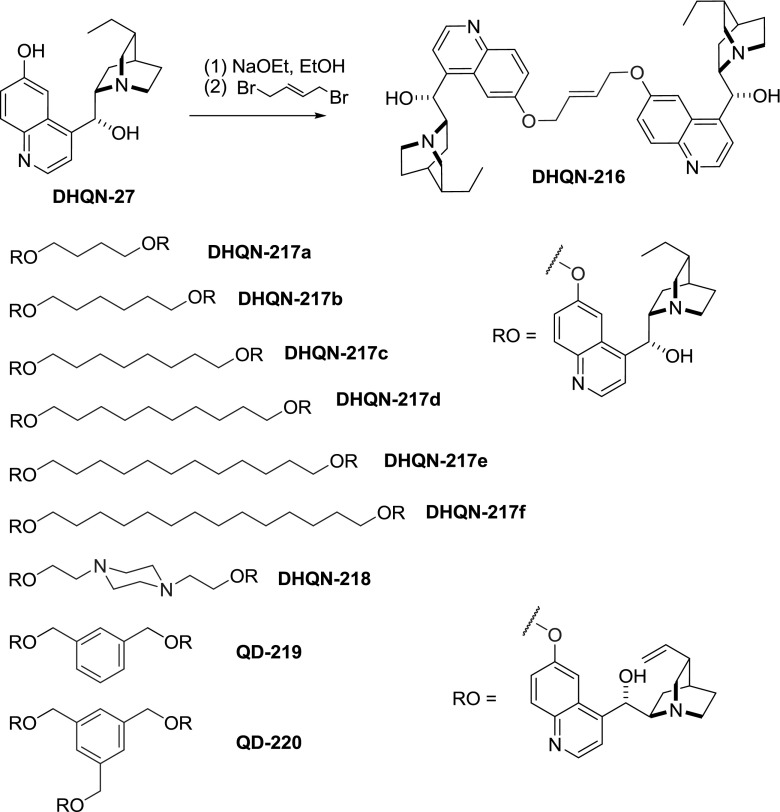
Dimeric and trimeric cupreine and cupreidine ethers

Quinoline and 6-methoxyquinoline rings also offer useful sites for modification. The $6'$-methoxy ether in quinine and quinidine can be cleaved either by HBr or with alkyl thiolates in DMF giving cupreine and cupreidine, respectively. The latter conditions are tolerant of the 10,11-double bond [142]. The acidity of the phenol group in cupreine (**QN-27**) and cupreidine (**QD-27**) facilitates a selective Williamson etherification. The reaction of dihydrocupreine salts with various $\upalpha , \upomega$-dihaloalkanes provided a library of compounds **DHQN-216**–**218** (Fig. 62). First dimers of this type were reported in the 1920’s and used 1,4-$E$-but-2-ene and butane linkers in **DHQN-216** and **DHQN-217a**, respectively [143]. Cowman obtained a series of dimers with linkers of 4-14 carbon atoms **DHQN-217a**–**f** as well as with piperazine-derived linker **DHQN-218** [144]. Cupreidine (**QD-27**) was also dimerized using $m$-xylylene dibromide into **QD-219** [66] and trimerized into **QD-220** with tris(bromomethyl)benzene [69].

**Fig. 63:**
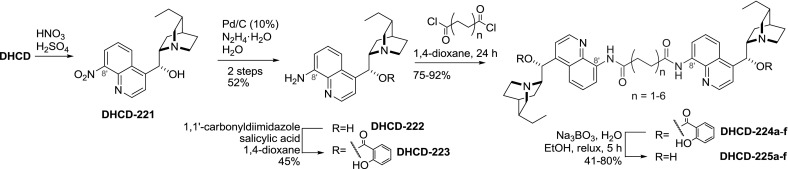
Synthesis of dimeric $8'$-anilides

Nitration of the quinoline ring of dihydrocinchonine and dihydrocinchonidine occurs preferentially at the $8'$ position. Subsequent reduction of the nitro group with hydrazine on palladium catalyst yielded primary aromatic amine **DHCD-222**. The reactions of $8'$-aminoalkaloid with several dicarboxylic acid chlorides yielded the corresponding dimers **DHCD-225a**–**f** in good to excellent yields. Similar reactivity of $8'$-amino and 9-hydroxy groups required prior use of a transient protecting group (i.e., salicylate) (Fig. 63) [144].

Both $6'$-ethers **DHQN-217a**–**f **and $8'$-amides **DHCD-225a**–**f **were assayed for inhibition of various strains of *Plasmodium falciparum*. *In vitro* tests showed that dimers containing an 8-methylene unit linker were most effective. The amide **DHCD-225d** (IC$_{50}$ 0.02–0.05$\,\upmu$M) was more active than ether **DHQN-217c** (IC$_{50}$ 0.08–0.26 $\upmu$M). Unfortunately, *in vivo* study of **DHCD-225d **revealed lower activity compared to chloroquine and pronounced toxicity [144].

**Fig. 64:**
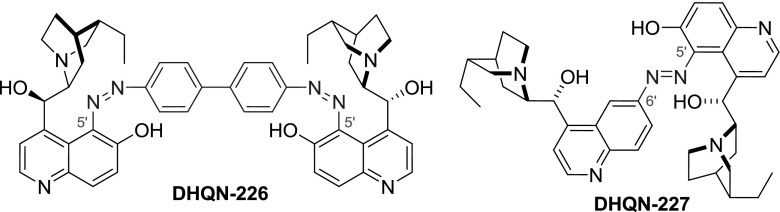
Dimeric diazo dyes

Electrophilic substitution in $6'$-metohxy- (**QN**, **QD**) and $6'$-hydroxyquinoline (**QN-27**, **QD-27**) occurs favorably at the $5'$-position. Susceptibility of cupreines to such aromatic substitution was exploited in the synthesis of alkaloid-derived dyes (Fig. 64). A dimeric dye **DHQN-226** was furnished in a reaction of cupreine sodium salt with bis-diazonium salt obtained from benzidine [145]. Also the phenol group in cupreine **DHQN-27** was converted to an amine and diazotized. The coupling of the alkaloid $6'$-diazonium salt with cupreine yielded a heterodimer **DHQN-227** [146].

**Fig. 65:**
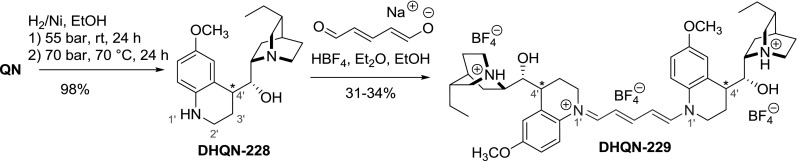
Synthesis of dimeric polymetine dyes

The $6'$-methoxyquinoline ring of *Cinchona* alkaloids was also partially hydrogenated to give tetrahydroquinoline. The reaction for both **QN** and **QD** led to mixtures of $4'$-epimers which were separated by 2–3 recrystallizations of mandelic acid salts (8–15 % yield for pure stereoisomers of **228**). The secondary amines **228** were then coupled with glutaconic aldehyde enolate to give polymetine dyes **229 **(Fig. 65). The dyes exhibited interesting chiral optical properties: markedly high specific rotation and maximum absorbance at 506–511 nm. The rotatory power was mostly dependent on the configuration at the $4'$ center, as exemplified by the $[\upalpha ]_{\mathrm{D }}(c 0.01, \hbox {EtOH})$ values of $-$1545 and +1135 for $(4'R)\text {-} \hbox { and } (4'S)$ **-DHQN-229**, respectively [147].

**Fig. 66:**
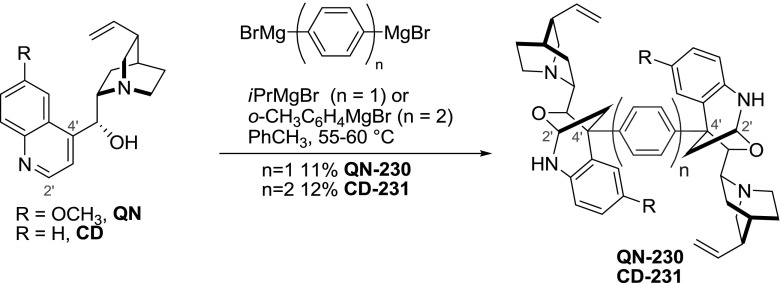
Formation of C$4'$-linked dimers

The quinoline ring of the alkaloids is also susceptible to Grignard reagents addition at the $2'$ and $4'$ positions. In non-etheral solvents the Grignard adds at the $4'$-position, and consecutively the deprotonated 9-hydroxyl group adds at the $2'$-position yielding a cyclic aminal with complete diastereoselection. The reaction gave fair yields (typically 65–35 %) for small and moderately bulky organomagnesium reagents. Consequently, dimers **QN-230** and **CD-231** were obtained in 11–12 % yield in a reaction of divalent 1,4-phenylene and 4,$4'$-biphenylene Grignard reagents, respectively (Fig. 66). The formation of monomeric products was avoided by using an excess of the less reactive organomagnesium reagents [148].

## Double-bridged dimers

**Fig. 67:**
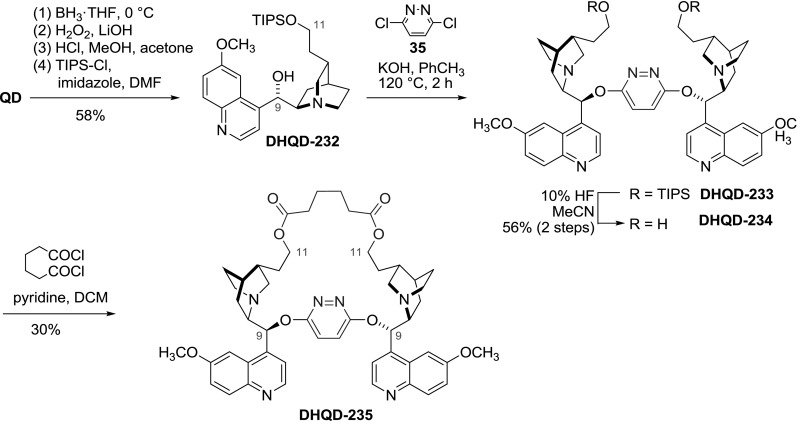
Synthesis of 9- and 11-tethered dimer

There are only few reported cases where the *Cinchona* alkaloid units are connected simultaneously at two distinct sites using two independent linkers. These include linear polymers **201**, **203** as well as copolymerized compound **10**. However, multiple connections between just two alkaloid units would result in a cyclic product of restricted conformation. In studies conducted to explain the stereoselectivity of the asymmetric dihydroxylation, two such structures **DHQD-235** and **DHQN-237** were described by the groups of Corey and Lohray, respectively. Both used a pyridazine linker attached with 9-ether bond and additionally tethered the dimer by either the vinyl group (**235**) [149] or the quinoline ring (**237**) [150]. The 9,11-tethered structure was prepared in a sequence of reactions starting from a Brown hydroboration of quinidine at the vinyl group, followed by protection of the primary 11-hydroxyl group with the triisopropylsilyl (TIPS) group. The protected alcohol **DHQD-232** was then coupled using dichloropyridazine (**35**), and the silyl ether was cleaved to form the dimeric 11-diol **DHQD-234**. In the last step, esterification of the dimer **DHQD-234** with adipoyl chloride gave the bridged dimer **DHQD-235**, as confirmed by X-ray, in a total of 9.7 % yield over 7 steps (Fig. 67) [149, 151].

**Fig. 68:**
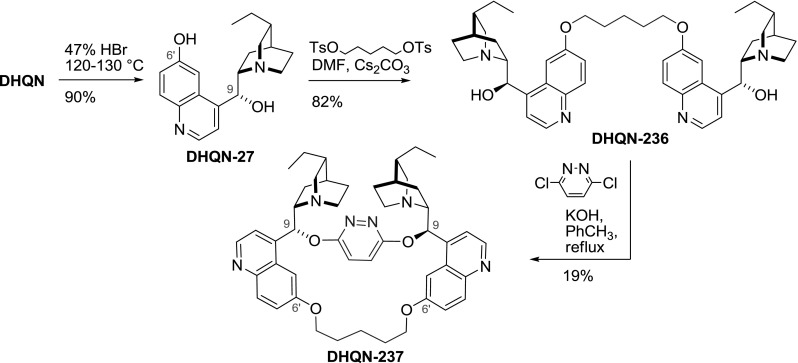
Synthesis of 9- and $6'$-tethered dimer

Lohray obtained cupreine **DHQN-27** by cleavage of the $6'$-methoxy ether. Then, reaction with 1,5-ditosyloxypentane gave the $6'$-tethered dimer **DHQN-236**. A subsequent reaction with dichloropyridazine (**35**) concluded the synthesis of **DHQN-237** in 14 % overall yield in 3 steps (Fig. 68) [150].

Asymmetric dihydroxylation reaction using 9,11-double tethered dimer **DHQD-235** provided enantioselectivity similar to AD with classic PYDZ-ligand (**DHQD-36**), and even outperformed it for some olefins. However, the additional $6'$-link in **DHQN-237** resulted in much lower level of enantioselectivity (32–72 %ee vs. 88–99 %ee).

**Fig. 69:**
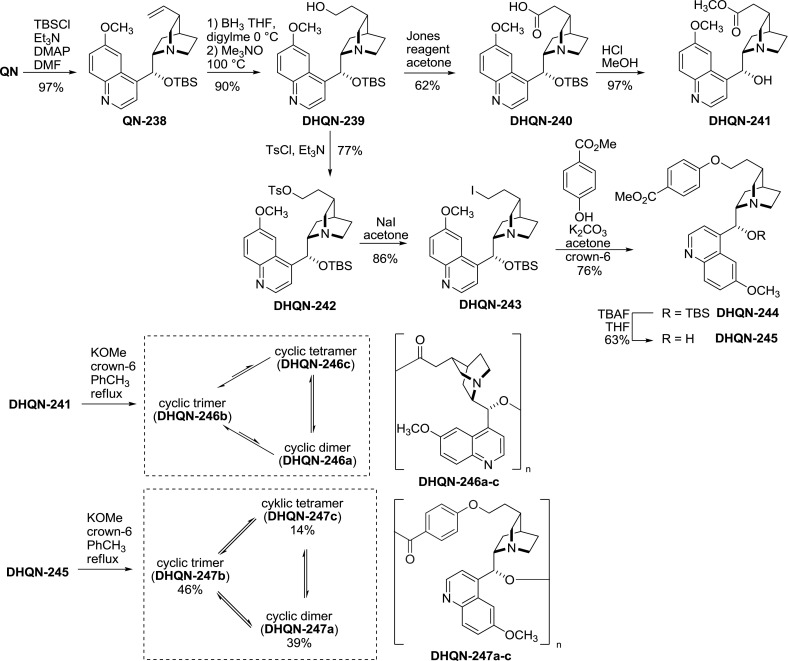
Synthesis of dynamic self-organizing libraries of cyclic oligomeric *Cinchona* alkaloids

Rowan and Sanders studied dynamic self-organization processes of modified *Cinchona* alkaloids possessing both a reactive ester and hydroxyl functionalities. Instead of careful stepwise tethering of the alkaloid molecules, they built a dynamic library of cyclic oligomeric alkaloids. They hydroborinated the 3-vinyl group, and the resulting 11-alcohol **239** was oxidized to the corresponding acid using the Jones reagent and then esterified to give **241** [152]. In an alternative approach, the 11-alcohol **239** was converted to a halide and etherified with methyl 4-hydroxybenzoate to give **245** [153]. The transestrification of **241** and **245** was carried with catalytic potassium methoxide and 18-crown-6. Under these conditions dynamic libraries of cyclic dimers, trimers, tetramers and acyclic products were formed (Fig. 69). The composition of these mixtures depended on both the linker and the alkaloid configuration. For quinine and cinchonidine-derived species, the cyclic trimer was the predominant (**QN-247b**, **CD-247b**) or nearly the exclusive product (**QN-246b**, **CD-246b**) that could be isolated upon crystallization [154]. In the case of quinidine, cyclic dimers were major products both with (**QD-247a**) and without a $p$-hydroxybenzoic spacer (**QD-246a**). In mixtures containing multiple types of precursors, the transesterification resulted in only small amounts of hybrid dimers and oligomers.

**Fig. 70:**
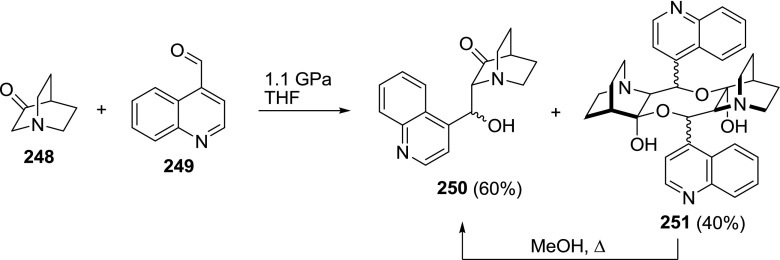
High pressure formation of dimeric hemiacetal

Experiments toward the total synthesis of the *Cinchona* alkaloids led to the formation of an unexpected dimer. One of the steps in Jankowski’s synthesis was the aldol condensation of 3-quinuclidinone (**248**) and quinoline-4-carbaldehyde (**249**). When this reaction was performed under high pressure conditions, in addition to the desired product **250**, a dimeric hemiacetal **251** was formed. The dimer **251** did not dissolve in any of the usual solvents, but heating a suspension in methanol resulted in a cleavage of the hemiacetal bonds to form hydroxyketone **250** (Fig. 70) [155]. The structure of **251** was suggested based on MALDI-MS and IR data, and the relative configuration was not determined.

## Closing remarks

There are many ways of combining multiple *Cinchona* alkaloid units into dimeric and oligomeric species, few avoid the use of linkers, and even highly hindered dimers can be formed. Most of the dimeric products were a result of planned stepwise syntheses of target molecules; nevertheless, methods of combinatorial chemistry were applied, including formation of dynamic libraries. The most exploited points of chemical diversity involve the central 9 position (Sharpless-type ligands and organocatalysts) and the quinuclidine N-1 nitrogen atom (phase transfer catalysts).

When compared to a monomeric analogue, the dimer has twice the number of reactive polar or electrically charged groups in close vicinity. The proximity of two bulky units also significantly restricts molecular conformation and may lead to formation of cavities. These factors are of principal significance in catalysis, and, in proper arrangement, have caused certain *Cinchona* alkaloid dimers to become some of the most effective asymmetric ligands and catalysts. A few of such products are now commercially available (e.g., **3**, **51**, **61**, **170**).

The dimeric *Cinchona* alkaloids are often more basic than their monomeric analogs and were more retained during their chromatographical isolation. Apparently, the same phenomenon seems to cause longer retention times on the solid phases modified with dimeric alkaloids.

In medicinal chemistry, the interaction of biomolecules with a tethered dimer may induce functional changes that could contribute to novel pharmacological properties. Few of the studies indicate that biological activity (cytotoxicity, transporter inhibition and antiplasmodial activity) can be found among the *Cinchona* dimers.
